# Endocytic Adaptor Proteins in Health and Disease: Lessons from Model Organisms and Human Mutations

**DOI:** 10.3390/cells8111345

**Published:** 2019-10-29

**Authors:** Domenico Azarnia Tehran, Tania López-Hernández, Tanja Maritzen

**Affiliations:** Leibniz-Forschungsinstitut für Molekulare Pharmakologie (FMP), 13125 Berlin, Germany; azarnia@fmp-berlin.de (D.A.T.); lopezhernandez@fmp-berlin.de (T.L.-H.)

**Keywords:** endocytosis, internalization, uptake, clathrin, neurotransmission, mouse, knockout

## Abstract

Cells need to exchange material and information with their environment. This is largely achieved via cell-surface receptors which mediate processes ranging from nutrient uptake to signaling responses. Consequently, their surface levels have to be dynamically controlled. Endocytosis constitutes a powerful mechanism to regulate the surface proteome and to recycle vesicular transmembrane proteins that strand at the plasma membrane after exocytosis. For efficient internalization, the cargo proteins need to be linked to the endocytic machinery via adaptor proteins such as the heterotetrameric endocytic adaptor complex AP-2 and a variety of mostly monomeric endocytic adaptors. In line with the importance of endocytosis for nutrient uptake, cell signaling and neurotransmission, animal models and human mutations have revealed that defects in these adaptors are associated with several diseases ranging from metabolic disorders to encephalopathies. This review will discuss the physiological functions of the so far known adaptor proteins and will provide a comprehensive overview of their links to human diseases.

## 1. Clathrin-Mediated Endocytosis

Cells are separated from their environment by a lipid bilayer membrane which prevents the unregulated entry of large molecules into the cell interior. However, they need to be in constant exchange with their surroundings to provide themselves with essential nutrients and to adequately respond to their environment. This is largely achieved via cell surface-localized proteins whose levels at the plasma membrane need to be tightly controlled to ensure their proper function. In addition, ligand-bound receptors and transmembrane proteins stranded at the plasma membrane after exocytosis have to be internalized. Both pathways are mediated by endocytosis, an elaborate process where the plasma membrane is invaginated and reshaped into a vesicle that finally detaches and moves inwards with its cargo.

**Figure 1:**
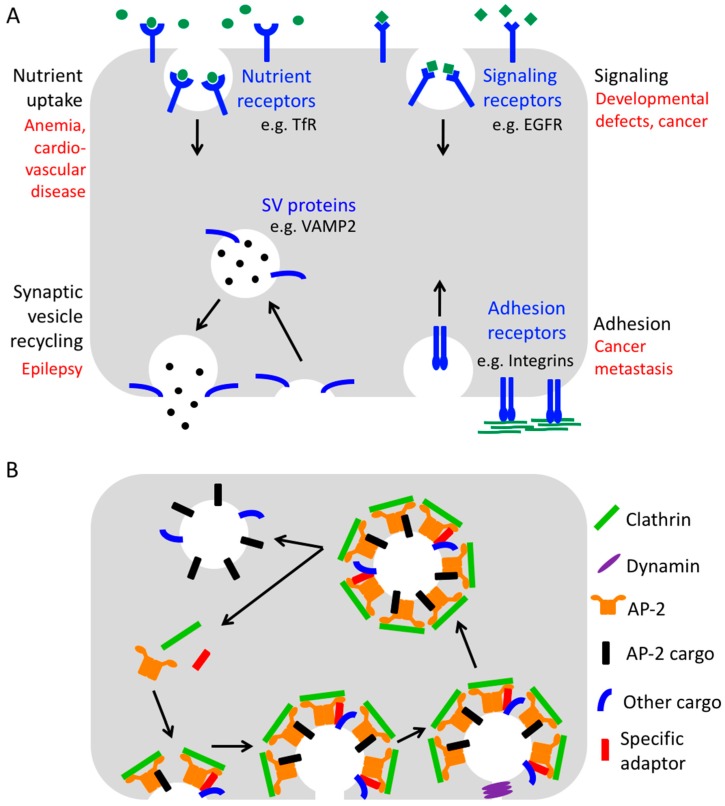
The process of endocytosis. (**A**) Physiological importance of endocytosis for various cellular pathways. Pathological consequences of endocytic defects affecting the different processes are depicted in red. (**B**) Simplified scheme of Clathrin-mediated endocytosis (**B**).

Consequently, endocytosis is in many ways crucial for human health (Figure 1A). For example, it is essential for the uptake of iron-loaded transferrin receptors (TfRs), which is a prerequisite for the generation of red blood cells. In addition, endocytosis allows the internalization of low-density lipoprotein (LDL)-loaded LDL receptors (LDLRs) providing cells with cholesterol and safeguarding serum cholesterol levels. It also controls the surface levels of hundreds of signaling receptors, thereby ensuring adequate signaling responses, and it regulates surface levels of adhesion receptors to enable proper cell adhesion and migration.

Finally, endocytosis plays an important role in the brain by facilitating synaptic vesicle recycling and by regulating the number of postsynaptic neurotransmitter receptors to maintain efficient neurotransmission and to support synaptic plasticity. Neurotransmission is based on the presynaptic release of neurotransmitters from synaptic vesicles which trigger rapid signaling cascades by binding to cognate receptors residing within the plasma membrane of the postsynaptic neuron. For the release of neurotransmitters, synaptic vesicles need to fuse with the presynaptic membrane. This fusion step requires a number of transmembrane proteins to be present on the vesicle such as the Ca^2+^ sensor synaptotagmin1 and the SNARE (soluble N-ethylmaleimide sensitive factor attachment protein receptor) protein Synaptobrevin2/VAMP2 (vesicle-associated membrane protein 2). Upon fusion, these proteins strand in the presynaptic membrane from where they must be efficiently retrieved by endocytosis and resorted onto a new set of synaptic vesicles to keep neurotransmission going.

Within the postsynaptic membrane, neurotransmitter receptors have to be present in exactly the right numbers since their amount strongly influences the postsynaptic response. In fact, the regulation of their surface level during synaptic plasticity is the mechanism underlying complex cognitive abilities such as learning and memory.

Understandably, defects in the endocytic machinery have been associated with numerous human pathologies including metabolic syndromes [1], cancer [2], psychiatric and neurodegenerative diseases [3]. Therefore, a detailed understanding of the function of endocytic proteins is crucial to dissect the molecular mechanisms of numerous human diseases and for designing novel therapeutic strategies for their treatment.

How is the endocytosis of transmembrane proteins achieved? The main route for the uptake of numerous cargo proteins and best-understood pathway is Clathrin-mediated endocytosis [4] (Figure 1B). This type of endocytosis derives its name from the protein Clathrin, a triskelion-shaped complex of three small (Clathrin light chain, ~25 kDa) and three large subunits (Clathrin heavy chain, ~190 kDa) which can assemble into flat and curved polygonal lattices and thereby “coat” the invaginating membrane bud starting with a Clathrin-coated pit and culminating in a Clathrin-coated vesicle [4]. Since Clathrin itself binds neither membranes nor cargo molecules, numerous accessory proteins are involved in its recruitment to endocytic sites.

**Figure 2:**
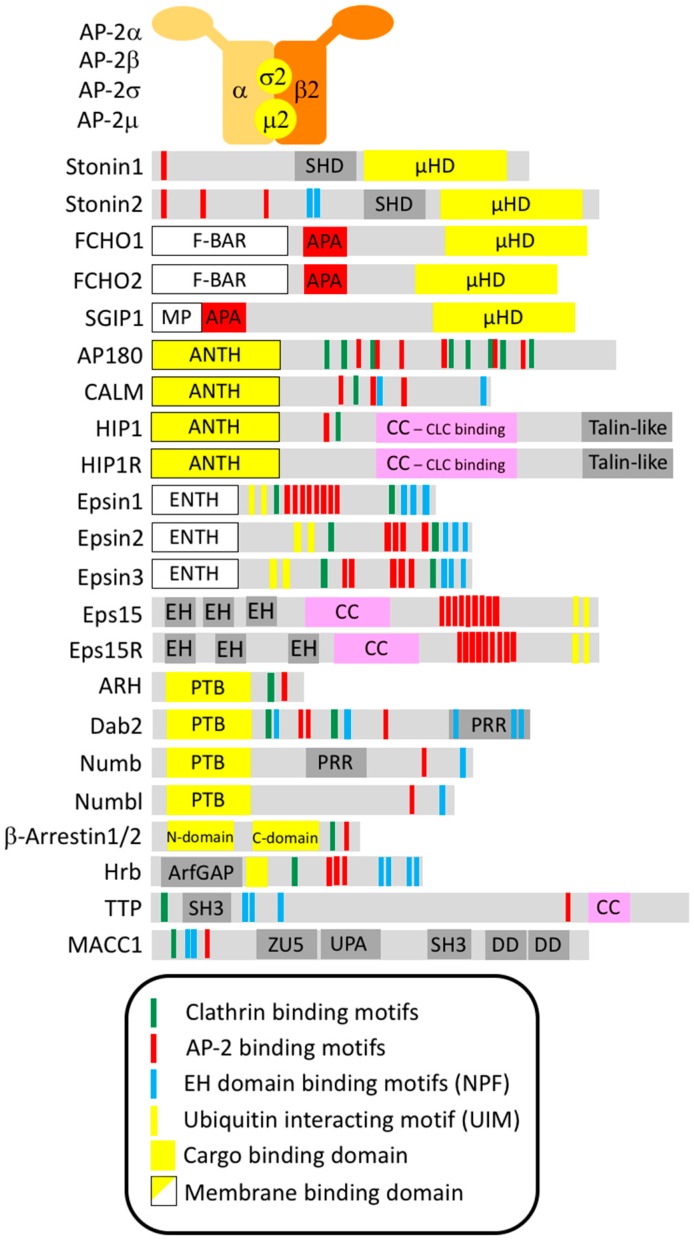
Domain structure of the known and proposed endocytic adaptors. Sketch of the domain structure of endocytic adaptor proteins including putative endocytic binding motifs. Domain structures were based on the following references: AP-2 [8], Stonin1/2 [25], FCHO1/2 and SGIP1 [26], AP180 and CALM [27], HIP1 and HIP1R [28], Epsin1/2/3 [29], Eps15 and Eps15R [30], ARH [31], Dab2 [32], Numb and Numbl [33], β-Arrestin1/2 [34], Hrb [35], TTP [36], MACC1 [37].

An important group, within the set of about 50 endocytic proteins, are the endocytic adaptor proteins. As reflected in their name, they serve as adaptors by connecting the endocytic machinery to the cargo proteins and in part also to the plasma membrane via phosphoinositide binding. The most important endocytic adaptor is the heterotetrameric AP-2 complex made up of the large α- and β2-adaptin subunits, the medium-sized μ2-subunit and a small σ2-subunit (Figure 2). Via its α/σ2 interface AP-2 can associate with cargos containing acidic cluster dileucine-based motifs adhering to the consensus sequence [DE]XXXL[LI] (X denotes any amino acid, and square brackets indicate that either amino acid can be present) [5,6,7]. Via its μ2-subunit AP-2 provides a binding site for transmembrane cargo proteins which harbor a tyrosine-based sorting signal in their cytoplasmic tails following the consensus motif YXXΦ (where Φ stands for an amino acid with a bulky hydrophobic side chain) [8,9,10]. In addition, the μ2-subunit also interacts with C2 domains as present in Synaptotagmin1 [11] or Otoferlin [12]. However, this leaves numerous important transmembrane proteins that have to be endocytosed unaccounted for, as for instance VAMP2 with contains none of the three sorting signals recognized by AP-2. Over the years, it became clear that AP-2 acts in conjunction with a range of additional adaptor proteins which recognize for instance short motifs, Ubiquitin moieties within ubiquitinated proteins or other folded domains, thereby extending the range of cargo proteins that can efficiently be linked to the endocytic machinery for Clathrin-mediated endocytosis.

The endocytic process starts with the binding of endocytic scaffold proteins such as the multi-domain protein Intersectin and adaptor proteins such as Eps15, Eps15R, AP-2, FCHO1/2 and CALM to the plasma membrane via their association with cargo proteins and/or the plasma membrane-enriched lipid phosphatidylinositol(4,5)bisphosphate [PI(4,5)P_2_] and/or each other [4]. This early arriving pioneer module recruits the Clathrin coat to the endocytic site and clusters cargo and coat components via a complex network of moderate to low affinity interactions which act cooperatively. Inbuilt regulatory mechanisms ensure the tight coupling between cargo selection and coat recruitment. For instance, the AP-2 complex needs first to bind to PI(4,5)P_2_ and to cargo which induces a large conformational change before it is able to efficiently recruit Clathrin [13].

The initial phase is followed by progressive membrane bending mediated by the coat and by BAR-domain containing proteins such as Endophilin and Amphiphysin leading to a constricted narrow neck as the last connection point between plasma membrane and the deeply invaginated vesicle-to-be. Relatively late it was recognized that not only in yeast [14,15], but also in mammalian cells under certain conditions [16], Actin polymerization at the endocytic site driven by the Actin nucleator ARP2/3 and its regulators like N-WASP is instrumental for endocytosis to proceed by contributing to membrane bending. To be effective, Actin polymerization has to be efficiently coupled to the Clathrin coat. Also during this step endocytic adaptor proteins play a pivotal role by linking Actin filaments to the Clathrin coat and to PI(4,5)P_2_. This is mainly done by members of the Epsin and HIP1R family, whose deletion consequently compromises endocytosis [4]. However, there are also important endocytic factors not belonging to the group of cargo adaptors such as FCHSD2 that contribute to the coordination of Actin dynamics with Clathrin-coated pit maturation [17].

The final scission of the invagination neck from the plasma membrane is brought about by an interplay between the BAR-domain proteins and the large GTPase Dynamin which forms an oligomeric helical collar around the neck and severs it upon a GTP-hydrolysis-dependent conformational change within the oligomer [4,18,19]. Finally Clathrin-mediated endocytosis terminates with the active disassembly of the Clathrin coat by the chaperone HSC70, recruited by the Clathrin interactor Auxilin, which allows vesicle fusion with the endosomal compartment [4,20,21,22]. For a more detailed mechanistic insight into the different endocytic steps we refer the reader to excellent reviews on the topic [4,8,23,24].

## 2. Endocytic Adaptors

Endocytic adaptors are crucial for linking select transmembrane proteins, that need to be internalized, to the endocytic machinery by recognizing cytosolic sorting determinants within the cargo proteins. They are mostly cytosolic proteins that are transiently recruited to endocytic sites where they cluster together with other coat components due to a cooperative network of low-affinity interactions. In line with their biological function, most endocytic adaptors have two “business ends”. On the one hand, they possess a cargo-binding domain for interaction with a specific endocytic sorting signal, on the other hand, they contain unstructured sequence stretches or flexibly connected small domains that harbor interaction surfaces for connecting to multiple endocytic proteins (Figure 2).

Accordingly, to be considered as a bona fide endocytic adaptor, a protein (1) has to bind at least one key player of the endocytic machinery, (2) has to recognize a cytosolic part of one or a set of transmembrane proteins and (3) has to facilitate the uptake of this/these proteins which are considered its “cargo”. In this article, we will discuss the current state of research for the bona fide endocytic adaptors that have been shown in mammals at the functional and structural level to fulfill all three criteria (e.g., AP180). In addition, we will also review putative adaptors (i) for whom it is not clear at the structural level how they interact with their cargo (e.g., HIP1), (ii) for which a cargo protein has only been identified in non-mammalian organisms (e.g., FCHO1/2) or (iii) which we regard as putative endocytic adaptors without identified cargo because of their sequence/domain similarity to established endocytic adaptors (e.g., Eps15R).

The great variety of transmembrane cargos requires the use of diverse sorting signals, ranging from linear motifs such as the YXXΦ sequence, which is bound by AP-2, to conformational determinants like the SNARE domain, which associates with AP180 and CALM, up to post-translational modifications such as ubiquitination, which is recognized by Eps15 for instance [8]. In addition, many endocytic adaptors have surfaces for the recognition of PI(4,5)P_2_ to associate with the plasma membrane. A subset also has interaction sites for the recruitment of Actin and Actin regulators to endocytic spots thereby contributing to membrane remodeling. For additional mechanistic insights on cargo recognition in Clathrin-mediated endocytosis we refer the reader to an excellent review on the topic [8].

In contrast to the loss of AP-2, the most general endocytic adaptor and also one of the central interaction hubs within the endocytic network, which severely compromises endocytosis, the loss of most of the additional adaptor proteins does not impair Clathrin-mediated endocytosis. Instead it primarily leads to the inefficient retrieval of their respective cargo proteins and thus to the accumulation of these cargo proteins at the plasma membrane. Consequently, the biological function and physiological importance of individual endocytic adaptor proteins depends on the function and relevance of their specific cargo proteins. In some cases the scenario becomes more complex since endocytic adaptors similar to many other proteins are increasingly recognized as fulfilling additional non-canonical roles [38].

The initial insights into the process of endocytosis and the function of endocytic adaptors were mostly derived from experiments on cultured cells revealing a wealth of mechanistic details, but shedding little light on how endocytosis affects diverse physiological processes and how its deregulation contributes to pathological conditions. For addressing these types of questions the analysis of animal models ranging from nematodes to mice has proven vital. In particular, the large collection of knockout (KO) mouse strains available today together with the advancing study of human disease mutations have proven invaluable tools for dissecting endocytic adaptor functions. Therefore, this review will put special emphasis on the phenotypes of the diverse adaptor KO mouse models as well as on what has been learned about the physiological importance of the different endocytic adaptors from pathological consequences of human mutations in these proteins. In the end we will also discuss how this information can open new avenues for therapeutic interventions.

### 2.1. AP-2

As outlined in the previous sections, AP-2 is not only a central interaction hub within the endocytic network, but also the specific cargo adaptor for a broad range of cargo proteins by recognizing dileucine and tyrosine-based motifs as well as C2 domains. Therefore, it is hardly possible to compile a comprehensive list of AP-2-dependent cargo proteins. However, to give a flavor of the diversity of the cargos of AP-2, we provide a selection of identified cargo proteins in Table 1.

While numerous proteins have been linked to AP-2, it is often unclear which of the sorting events are physiologically particularly relevant in any given tissue. We will here discuss what has been learned from pathological consequences of defects in AP-2-dependent sorting and refer the reader for more details on the molecular mechanism of cargo recognition by AP-2 to an excellent review on this topic [8].

#### 2.1.1. Lessons from AP-2-Deficient Mouse Models

Hardly surprising, the ubiquitous loss of AP-2 is not compatible with life. Mice carrying constitutive deletions of either the AP-2μ [39] or the AP-2β subunit [40] die at early embryonic respectively perinatal stages underlining a crucial requirement for AP-2 during mammalian development. Furthermore, disruption of AP-2β in mice also causes non syndromic cleft palate, a craniofacial malformation [40], however the molecular basis of this developmental defect is presently unclear. For AP-2σ, no KO mouse model has been reported so far. However, a mutagenesis screen in mice revealed a deletion of 17 conserved amino acids in the *AP2S1* gene. While mice carrying the deletion in a heterozygous manner did not show any alterations, homozygous carriers suffered from embryonic lethality [41].

The lethal phenotype caused by the complete loss of AP-2 limits the possibility of investigating the role of this multimeric adaptor in vivo and thus called for the generation of tissue-specific KO mice. Neuron-specific AP-2μ KO mice were born at a lower than expected ratio, did not thrive and finally died after ~3 weeks. Consistent with this, histology revealed severe neurodegeneration of thalamus and cortex as well as reduced neuronal complexity, implicating AP-2 in the prevention of neuronal loss and neurodegeneration [38]. The mechanism behind the reduced neuronal complexity appears to be a non-canonical role of AP-2 in the retrograde transport of BDNF (brain-derived neurotrophic factor)/TrkB (tropomyosin receptor kinase B)-containing autophagosomes. Thereby, AP-2 promotes BDNF/TrkB signaling and thus neuronal branching and survival [38]. A second tissue-specific mouse model targeted AP-2µ in cochlear inner hair cells to investigate the role of AP-2 at the extremely fast releasing inner hair cell synapse which is critical for hearing [12]. Indeed, mice with inner hair cell-specific deletion of AP-2µ displayed a profound hearing impairment accompanied by a mislocalization and partial loss of Otoferlin [12], a C2 domain protein proposed to act as Ca^2+^ sensor for the release of synaptic vesicles in inner hair cells. Biochemical experiments in fact identified Otoferlin as a novel AP-2 cargo protein [12]. By sorting Otoferlin AP-2 appears to promote rapid release site replenishment at inner hair cell synapses.

Little is known about the consequences of AP-2 loss in non neuronal tissues. However, AP-2 very likely also has crucial and specific functions in other cell types, for instance in glial cells which are as important as neurons for brain function. Many studies have shown that defective astrocyte function might contribute to neuronal dysfunction, thus playing a complex role in the pathogenesis of neurodegenerative and neurological diseases [42]. Thus, clearly more tissue-specific KO approaches are needed to identify the most important physiological cargos and functions of AP-2 in different cell-types.

#### 2.1.2. Lessons from Human Mutations in AP-2 Subunits

Considering the requirement for AP-2 for viability, human loss of function mutations are not to be expected. However, milder mutations in the genes encoding AP-2σ (*AP2S1*) and AP-2µ (*AP2M1*) have been identified as cause for familial hypocalciuric hypercalcemia (FHH) type 3 [43] respectively epileptic encephalopathy [3]. FHH is an autosomal dominant disorder characterized by elevated serum Ca^2+^ concentrations in association with low Ca^2+^ levels in urine. It can also be associated with slightly elevated circulating levels of parathyroid hormone (PTH) and mild hypermagnesemia [44]. FHH type 1 and 2 are usually asymptomatic. FHH type 3 is considered a more severe FHH variant [45] showing increased serum PTH concentrations, hypophosphatemia, osteomalacia and cognitive dysfunction [43]. In all three forms of FHH the suggested pathophysiological mechanism involves impaired Ca^2+^ sensing. Interestingly, all the known *AP2S1* mutations causing FHH type 3 affect Arg15, an amino acid that directly contacts the dileucine-based internalization signal present in the cytoplasmic tail of the Ca^2+^ sensing G protein-coupled receptor (GPCR) CaSR. In line with this, all three missense mutations identified in *AP2S1* cause CaSR to be less efficiently internalized. This defective internalization is likely causative for the disease since mutations in *CASR* underlie FHH type 1 [43,45]. A recent analysis of more than 11,000 exome datasets aimed at finding new AP-2σ variants underlying FHH3 identified eight novel AP-2σ variants predicted to either disrupt polar contacts within the AP-2σ subunit or to affect the interface between the AP-2σ and AP-2α subunit. Functional studies revealed that three of them lead to abnormalities in CaSR-mediated signaling, thus, also mutations in *AP2S1* which do not involve Arg15 may alter CaSR function [46].

A de novo missense mutation in *AP2M1* (p.Arg170Trp) was recently identified in a comprehensive approach based on whole exome sequencing data of a cohort of 314 individuals suffering from epileptic encephalopathies paired with an analysis of their phenotypic similarities [3]. Arg170 is part of a region within AP-2µ that is proposed to stabilize the open conformation of AP-2 which allows cargo binding. Molecular modeling of AP-2 suggested that the mutation might therefore impair cargo recognition by AP-2. In agreement with this, AP-2 complexes comprising the mutated AP-2µ subunit were less efficient at Transferrin uptake [3]. How exactly this defect causes an epileptic phenotype remains unclear. However, epilepsy is generally viewed as the result of an imbalance between excitatory and inhibitory neurotransmission, and it is known that other endocytic adaptors like AP180 cause epileptic seizures in mice by missorting synaptic vesicle proteins and thereby especially impairing inhibitory neurotransmission (Section 2.4). Since AP-2 is not only involved in the sorting of synaptic vesicle proteins, but also regulates the surface expression of postsynaptic neurotransmitter receptors, it is easy to envisage how defects in AP-2-dependent sorting might cause epilepsy. However, the number of AP-2-dependent cargos will make it extremely difficult to pinpoint a single culprit. In fact, small changes in the effectiveness of AP-2-mediated sorting of different cargos might in total lead to large differences in the excitability of the neuronal network, thereby elevating seizure susceptibility.

#### 2.1.3. Additional Links of AP-2 to Human Disease

Alterations in AP-2 might also be involved in other neurological diseases, because not only *PICALM*, as discussed in Section 2.4, but also *AP2A1* and *AP2A2* were identified as gene loci that are linked to Alzheimer’s disease, emphasizing the association of cellular trafficking pathways with Alzheimer’s disease susceptibility [47], even though the exact role of *AP2A1* and *AP2A2* in Alzheimer’s disease is not clear yet. As discussed also in later sections, many endocytic adaptors have links to cancer due to the aberrant signaling that is caused by their misregulation. In line with this, a recent study using quantitative proteomics revealed that AP-2α, AP-2β and AP-2σ and also other endocytic factors exhibit decreased expression levels in gliomas [48]. In addition to effects via its endocytic function, AP-2 could also modulate tumor cell migration and invasion by its non-canonical role in microtubule acetylation as recruiter of α-Tubulin Acetyltransferase (αTAT1) which affects directional cell locomotion and chemotaxis [49].

AP-2 does not only have cellular cargos, but is also involved in the uptake of a number of viruses such as hepatitis C virus (HCV) [50] or human immunodeficiency virus type 1 (HIV-1). AP-2 is proposed as the critical adaptor selected by the HIV accessory proteins Vpu and Nef to mediate HIV endocytosis [51]. The C-terminal loop of Nef contains for instance an ExxxLΦ motif that directs the AP-2-dependent endocytosis of CD4, the primary receptor for HIV entry into T cells [52]. Thus, not only defects in AP-2-dependent internalization are relevant to disease, but also the hijacking of the endocytic machinery by viruses.

Finally, the misdirection of AP-2-dependent sorting towards cargos that are not internalized by AP-2 under physiological conditions can also have pathogenic consequences. There is in fact a set of diseases caused by missense mutations affecting intrinsically disordered protein regions (IDRs) whose pathological mechanism mostly remains enigmatic. An elegant work using a proteomic screen to investigate the impact of mutations in IDRs shows that some of these disease-associated mutations result in dileucine motifs causing AP-2-dependent endocytosis of the affected proteins [53]. One example are mutations in the glucose transporter GLUT1/SLC2A1 which cause GLUT1 deficiency syndrome. Consistent with the identified novel dileucine motif, the mutant GLUT1 mislocalized to intracellular compartments, a phenotype that could be rescued by depleting AP-2 [53]. Further work is needed to dissect the extent of “dileucineopathies” among the diseases caused by mutations in IDRs.

### 2.2. Stonin1 and Stonin2

Proteins of the Stonin family are monomeric endocytic adaptors containing a domain of high sequence similarity to the µ2 subunit of AP-2. Consequently, this domain has been named µ-homology domain (µHD). In fact, the µHD of human Stonin2 is ~30% identical to the cargo-binding C-terminus of the AP-2 µ2 subunit [8]. Thus, it was early on suspected to serve in cargo recognition. In addition to the µHD, the Stonins contain an unstructured N-terminus harboring a variable number of WxxF motifs for binding to AP-2. Stonin2 also contains NPF (asparagine-proline-phenylalanine) motifs for interacting with the EH (Eps15 homology) domains of the endocytic proteins Eps15 and Intersectin which further underscored the notion that Stonins function as endocytic adaptor proteins [25]. The unstructured N-terminus is followed by the Stonin homology domain (SHD) which is conserved in all Stonins, but still has no assigned function (Figure 2).

#### 2.2.1. Stonin2 as a Sorter for Synaptotagmin1

The Stonins were first analyzed in D. melanogaster which possesses only one Stonin variant called StonedB which is functionally related to Stonin2. The Stonins derive their name from the fact that temperature-sensitive mutants in Drosophila are paralyzed i.e., “stoned” [54,55]. This suggested an important function of Stonins within the nervous system. In fact, synapses of mutant larvae exhibited defects in the recycling of synaptic vesicles, a reduced synaptic vesicle pool and a severe mislocalization of Synaptotagmin1 thereby suggesting it as a potential cargo for the putative endocytic adaptor StonedB [56,57]. Synaptotagmin1 as the major Ca^2+^ sensor on synaptic vesicles is crucial for synchronous neurotransmitter release and thus essential for life [58]. Given its essential function in the Ca2^+^-dependent release of synaptic vesicles, Synaptotagmin1 needs to be efficiently recycled upon vesicle fusion in order to regenerate a new set of fusion-competent synaptic vesicles and thereby maintain neurotransmission. Yet, Synaptotagmin1 does not contain any of the typical motifs for binding to AP-2 [59,60]. However, it can interact with AP-2 via a sequence of basic amino acids within its C2B domain. But this interaction is apparently too weak to mediate on its own the endocytosis of Synaptotagmin1, indeed, it is not efficiently internalized when ectopically expressed in non neuronal cells [61,62] suggesting the existence of an extra adaptor protein for its uptake. In fact, the embryonic lethality of StonedB mutants could be reversed by overexpressing Synaptotagmin [56] further implicating StonedB as its sorting adaptor. In addition, in non neuronal cells, the overexpression of Stonin2 was sufficient to achieve the internalization of Synaptotagmin1 [63]. Finally, a direct interaction between Stonin2 and Synaptotagmin1 was demonstrated [63,64,65,66] revealing that it is indeed the µHD of Stonin2 which binds its cargo Synaptotagmin. The sorting motif within Synaptotagmin1 proved to be clusters of basic residues, mostly located on the surface of its C2A domain [11].

#### 2.2.2. Lessons from Stonin-Deficient Mouse Models

While the loss of the StonedB homolog Unc41 in Caenorhabditis elegans also leads to a severe paralytic phenotype [11], it came as a surprise that the KO of Stonin2 and also the double KO of Stonin1 and Stonin2 in mice caused only mild behavioral alterations such as increased exploration [67]. Still, these mice showed as expected a partial mislocalization of Synaptotagmin1, but not of other synaptic vesicle proteins, to the presynaptic membrane confirming also for mammals that Stonin2 acts in Synaptotagmin1 sorting [67]. However, there appeared to be a second sorting factor for Synaptotagmin1 in mammals. In fact, later the synaptic vesicle protein SV2, which belongs to a protein family which is conserved in vertebrates, but not in invertebrates, was proven to be this factor. While SV2 is crucial for life and an important target for anti-epileptic drugs, its molecular function is still largely unclear (see [68] for a recent review). However, SV2 binds Synaptotagmin1 [69,70,71], and studies on SV2A/B double KO mice revealed a mislocalization of Synaptotamin1 to the neuronal surface [72]. Experiments with Stonin2/SV2A/SV2B triple KO mice finally confirmed that these proteins have a partially redundant role in Synaptotagmin1 sorting in mammals since the Synaptotagmin1 mislocalization and the downstream defects in neurotransmission were stronger in the triple mutant than in the single KO lines [73]. This underlines that not only bona fide cytosolic endocytic adaptors assist in the retrieval of transmembrane proteins, but also integral membrane proteins which are assumed to have a different primary function. Additional examples are the facilitation of VAMP2 sorting by the transmembrane synaptic vesicle protein Synaptophysin [74,75] in addition to AP180/CALM (see also Section 2.4) and the internalization of AMPA-type glutamate receptors by Synaptotagmin3 in addition to AP-2 [76]. This highlights also a second concept: In an increasing number of cases, it is more than one adaptor protein that contributes to the efficient sorting of a specific protein (see also Section 3).

While the role of Stonin2 in neuronal cells has been established, its function in non neuronal cells is still unclear. Likewise, we are only beginning to understand the function of its mammalian family member Stonin1. Stonin1 greatly resembles Stonin2 in its domain structure, but is much less prominently expressed in brain. Instead, it is for instance well expressed in fibroblasts which lack Stonin2. In this cell type the most intriguing finding so far is the impact of Stonin1’s deletion on the morphology and turnover of focal adhesions [77]. Indeed, loss of Stonin1 leads to smaller focal adhesions, but it is not clear yet whether/how this phenotype is related to the endocytic adaptor function of Stonin1. Surprisingly, Stonin1 KO mice have no discernible phenotype so far arguing for compensatory mechanisms in vivo [77].

#### 2.2.3. Links of Stonin Proteins to Human Disease

There are no known disease mutations in Stonin genes to date, however the Stonin2 gene *STON2* lies within a chromosomal region that has been associated with Tourette syndrom [78] and has also been linked to schizophrenia in one study [79] underlining the potential importance of Stonins for brain physiology in humans. Furthermore, the expression level of Stonin1 was found altered in glioma biopsies [48].

### 2.3. FCHO1, FCHO2 and SGIP1

FCHO1, FCHO2 and SGIP1 also possess a µHD located at their C-terminus [26]. However, their µHD differs from the Stonin µHD in not only binding cargo proteins, but in also connecting to endocytic adaptors such as Eps15 [26,80] and Dab2 [81]. Besides, it shows less than 20% sequence identity to the µ2 subunit of AP-2 and, therefore, it was harder to identify [26]. This led to the naming of FCHO1 and FCHO2 as “FCH domain only” for their N-terminal extended FCH domain (nowadays better known as F-BAR domain) since the homology to µ2 had originally been overlooked [82] (Figure 2). After the identification of the µHD, the protein family was termed “Muniscins” deriving from µHD and meniscus for their crescent-shaped membrane binding F-BAR domain [26], which also promotes their oligomerization [83]. However, the third Muniscin family member, the brain enriched SGIP1 [SH3 domain growth factor receptor-bound 2-like (= Endophilin) interacting protein 1] and its splice variant SGIP1α which derive their name from their interaction with Endophilin, does not contain an F-BAR domain, but instead harbors a membrane phospholipid binding (MP) domain [84] (Figure 2).

#### 2.3.1. Molecular Functions of Muniscins

The FCHOs are believed to be critical components of Clathrin-mediated endocytosis since their knockdown impaired the uptake of a range of cargos comprising Transferrin, EGF and LDL [85,86]. While the original idea of FCHOs as essential nucleators of Clathrin-coated pits [85] was challenged by later studies [87] showing the independent arrival of AP-2 at these structures [88], evidence has accumulated that FCHOs promote Clathrin-mediated endocytosis via different mechanisms: By binding to early coat components such as Eps15 and Dab2, they foster the assembly of the early arriving pioneer module. Via their AP-2 activator domain (APA) and in complex with Eps15, they promote the transition of AP-2 to its open conformation [89,90,91]. With their F-BAR domain they contribute to curvature sensing and membrane remodeling [85]. In this manner FCHOs help to stabilize Clathrin-coated pits and facilitate their maturation.

While there is still no cargo identified for FCHO1 and FCHO2 in mammalian cells, SGIP1α was recently identified as specific sorting adaptor for Synaptotagmin1 at hippocampal synapses. Like Stonin2 SGIP1α binds to Synaptotagmin1 via its µHD and facilitates its internalization by linking it to the endocytic machinery [92]. Even though SGIP1α shares the µHD as cargo recognition site with Stonin2, this finding came as a surprise since with Stonin2 and SV2A/B already two redundant sorting mechanisms for Synaptotagmin1 had been identified (discussed in Section 2.2). Apparently, the high-fidelity sorting of the crucial Ca^2+^ sensor Synaptotagmin1 is ensured by multiple independent adaptor interactions.

SGIP1 was also shown to interact with the cannabinoid receptor CB1R which plays an important role in the modulation of synaptic plasticity. However, currently the physiological consequences of this interaction remain enigmatic [93].

#### 2.3.2. Insights from Muniscin-Deficient Model Organisms

The first implications of Muniscins as endocytic adaptors came from studies of the yeast homologue SYP-1 which was found to colocalize with endocytic proteins and to be essential for the specific internalization of the stress sensor Mid2 [26]. Also studies in zebrafish identified an endocytic cargo of FCHO proteins, the BMP (bone morphogenetic protein) receptor Alk8 [87]. The deregulation of BMP signaling in FCHO-deficient zebrafish causes developmental defects. However, as discussed in the preceding paragraph, in mammals it is presently unclear whether FCHO1 and FCHO2 have an important role as cargo adaptors since no specific cargos have been identified, while SGIP1α was identied as adaptor for Synaptotagmin1.

There are no publications on mouse models for FCHO1/2 and SGIP1 yet. However, the IMPC (International Mouse Phenotyping Consortium) reports complete preweaning lethality for an FCHO2 KO mouse strain (FCHO2 KO phenotyping data. Available online: https://www.mousephenotype.org/data/genes/MGI:3505790) arguing for an important role in mammals. The mouse model referenced for SGIP1 at the IMPC is viable, however displays abnormal behaviour and a cardiovascular phenotype in line with a neuronal and potentially cardiac role of SGIP1 (SGIP1 KO phenotyping data. Available online: https://www.mousephenotype.org/data/genes/MGI:1920344).

#### 2.3.3. Links of Muniscin Proteins to Human Disease

For FCHO1, data about human mutations was recently published. These mutations associate with a combined immunodeficiency that causes recurrent infections. Based on experiments with T cells derived from a single patient, the authors show decreased Transferrin uptake and impaired T cell proliferation [94].

SGIP1 was initially identified in a screen for central nervous system regulators of energy balance and obesity, and it was found upregulated in the hypothalamus of an obese mouse line [95]. Its suppression decreased body weight in obese mice via reduced food intake [95]. In line with this, SGIP1 was associated with measures of obesity such as fat mass in humans [96,97]. Besides, it was linked to alterations in EEG and ECG characteristics and to alcoholism. However, these studies require further replication to be conclusive [98,99,100,101].

### 2.4. AP180 and CALM

More than 30 years ago AP180 (assembly protein 180, encoded by the gene *SNAP91* and in the beginning also known as AP3, NP185, F1-20 and pp155) was identified as a highly abundant component of Clathrin-coated vesicles using bovine brain extract [102,103]. While there is a single homologous protein in D. melanogaster (Lap) and in C. elegans (Unc11), yeast and mammalian genomes encode two family members, called YAP1801 and YAP1802 in yeast and AP180 and CALM (encoded by the gene *PICALM*) in mammals. CALM was originally discovered as a gene fusion with the transcription factor AF10 in certain leukaemias leading to its name "Clathrin-assembly lymphoid myeloid leukaemia gene” [104]. AP180 expression is mostly restricted to neurons and only detected at the presynapse [105], while CALM has been detected in many tissues [104,106], including neurons where it is found pre- and post-synaptically [107] suggesting that, in contrast to AP180, it may regulate distinct endocytic events in different cell types and within the two synaptic compartments.

Both AP180 and CALM fulfill the definition of monomeric adaptor proteins consisting of a cargo binding module, their higly conserved ANTH (AP180 N-terminal homology) domain which simultaneously binds PI(4,5)P_2_ [108], and a disordered C-terminal region harbouring a variable number of Clathrin-, AP-2- and EH-domain binding motifs [27] (Figure 2). Early in vitro studies demonstrated that AP180 promotes the assembly of Clathrin lattices on lipid bilayers and is needed for obtaining homogenously sized small Clathrin-coated vesicles [109]. This suggested that AP180 is a critical Clathrin assembly factor which helps to arrange Clathrin triskelia into properly shaped vesicle coats. In addition, CALM was later shown to harbor an N-terminal amphipathic helix whose membrane insertion promotes endocytic membrane remodeling [110]. However, studies in mammalian cell lines yielded ambiguous results and overall did not support an essential role for AP180/CALM in endocytosis. Even though RNAi-based depletion of CALM in HeLa cells led to larger and more irregularly formed Clathrin-coated pits [111,112], Transferrin uptake was either normal [111] or only partially affected [110]. Also the analysis of AP180/CALM deficient mouse embryonic fibroblasts revealed only modest effects on Clathrin-coated pit dynamics [113]. These experimental data led to the hypothesis that, even though AP180/CALM are together with Clathrin and AP-2 the most abundant components of Clathrin-coated vesicles [110], they are not essential for general endocytosis.

#### 2.4.1. Studies of AP180 and CALM in Non-Mammalian Model Organisms

However, AP180 and CALM serve important functions as cargo-specific adaptors which were mostly discovered thanks to the analysis of different model organisms lacking these proteins. In line with the non essential role of AP180/CALM in endocytosis, loss of Yap1801/Yap1802 in yeast did not affect Clathrin-mediated endocytosis in general, but specifically altered the localization of the VAMP homologue Snc1 [114] pointing to a role of AP180/CALM in the trafficking of SNARE proteins of the VAMP family. In agreement with the yeast studies, AP180 deficient D. discoideum displayed a mislocalization of VAMP7B leading to an enlarged contractile vacuole due to excessive homotypic fusion [115]. In C. elegans the loss of Unc11 caused the mislocalization of the synaptic vesicle protein VAMP2 to the neuronal plasma membrane and an accumulation of large vesicles at presynaptic boutons, underscoring the pivotal role of AP180/CALM in sorting VAMPs and in regulating the generation of synaptic vesicles [116,117]. Moreover, Unc11 was shown to be crucial for the endocytosis of postsynaptic ubiquitinated glutamate receptors [118]. However, the molecular mechanisms underlying this process are still not clear, and it has not been addressed whether this function of AP180/CALM is conserved at mammalian synapses. In flies the most striking consequences of Lap loss were a reduction in synaptic vesicle density, an increased number of endosomal-like vacuoles and, in contrast to the specific sorting defects of other model organisms, a mislocalization of diverse neuronal proteins such as Synaptotagmin, Intersectin, and glutamate receptors leading to defective neurotransmission [119,120].

#### 2.4.2. Lessons from AP180- and CALM-Deficient Mouse Models

An AP180 deficient mouse model later confirmed the importance of AP180/CALM for neurotransmission also for mammals. Neurons derived from AP180 KO mice displayed a selective accumulation of VAMP2 at the neuronal surface which was further aggravated by concomitant depletion of CALM arguing for its redundant function in VAMP2 sorting at the presynapse [105]. In line with the importance of VAMP2 as fusion protein for the release of neurotransmitters from synaptic vesicles, VAMP2 missorting in AP180 KO mice led to defects in neurotransmission, epileptic seizures and premature death [105].

The specific endocytic sorting of VAMP2 is mediated by direct binding between the AP180/CALM ANTH domain with the N-terminal half of the VAMP2 SNARE helix [121,122]. Biochemical and cell biological studies demonstrated that CALM can also sort other VAMP isoforms including VAMP3, 4, 7 and 8 [110,112], indicating the universality of CALM–VAMP interactions and highlighting the potential importance of CALM in the regulation of other types of membrane fusion events. However, the physiological relevance of these additional interactions is presently largely unclear apart from the fact that missorting of VAMP3 and VAMP8 was linked to defects in autophagy [123]. Instead, the analysis of CALM-deficient mouse models [113,124] revealed first of all an essential role for CALM in the sorting of the TfR, specifically in red blood cell precursors, even though the molecular basis for this interaction and its cell-type specificity is not resolved yet. Thus, constitutive CALM KO mice suffer from embryonal or early postnatal lethality, presumably due to deficient Transferrin uptake in erythroid cells and a resulting anemia [113,124].

While the role of AP180 in neurons appears to be largely elucidated, non-redundant functions of CALM, especially at the postsynapse, have not been addressed yet. An extensive characterization of CALM in neurons based on neuron-specific CALM KO mice and AP180/CALM double KO (DKO) mice will help to decipher the contribution of CALM to neuronal physiology and will open new avenues for identifying potential additional cargo proteins.

#### 2.4.3. Links of AP180 and CALM to Human Disease

Despite the severe defects observed in AP180 KO mice, there are no disease-causing human mutations known. Instead, AP180 has only been vaguely associated with psychotic bipolar disorder [125] and Autism spectrum disorders [126]. In contrast, CALM is one of the few firmly established risk factors for late onset Alzheimer’s disease [127]. In fact, its most reproducible SNP r3851179 is currenctly one of the top 6 risk sites for Alzheimer’s disease in the AlzGene database (AlzGene database. Available online: http://www.AlzGene.org). This SNP is located in a non-coding region of *PICALM* and likely influences the expression level of CALM [128]. While CALM is clearly associated with Alzheimer’s disease, there appear to be different ways in which it might modulate the disease.

Alzheimer’s disease is characterized by the presence of extracellular toxic plaques of amyloid β (Aβ) peptides, proteolytic fragments of the amyloid precursor protein (APP). These peptides cause progressive synaptic dysfunction, neuronal atrophy and a fast decline in memory. The best supported hypothesis for the influence of CALM on Alzheimer’s disease pertains actually not to neurons, but to endothelial cells which can clear Aβ from the brain. CALM promotes this process by facilitating the uptake of the Aβ-bound LRP1 protein, an important Aβ clearance receptor [128]. iPSC (induced pluripotent stem cell)-derived endothelial cells carrying the protective rs3851179^A^ variant had ca. 75% higher levels of CALM mRNA and showed enhanced Aβ clearance [128]. In contrast, the effect of neuronally expressed CALM on Alzheimer’s disease is much more controversial. On the one hand, CALM appears to serve as endocytic adaptor for the uptake of APP, potentially in conjunction with other adaptors such as Numb [129], thereby modulating the trafficking and proteolytic processing of APP [130] and increasing the plaque load in an Alzheimer’s disease mouse model upon CALM overexpression [130]. On the other hand, it seems to facilitate the uptake of Nicastrin, one of the proteins involved in the proteolytic processing of APP, and a reduction of CALM in vivo decreased Aβ levels [131]. Besides, by impacting the sorting of different VAMPs CALM could also indirectly affect the trafficking itineraries, processing and degradation of proteins relevant for Alzheimer’s disease [123]. Finally, in yeast and C. elegans CALM was able to reduce Aβ oligomerization by an enigmatic mechanism [132,133]. It is presently unclear whether all these suggested Alzheimer’s disease relevant functions of neuronal CALM actually contribute equally to its effect on Alzheimer’s disease and whether the neuronal and endothelial effects of CALM are both protective or rather at odds. Further studies involving neuron-specific CALM KO mice are needed to resolve these issues.

#### 2.4.4. Non-Canonical Roles of CALM

Several studies have suggested that CALM might have a function at endosomal compartments in addtion to its canonical role in endocytosis [111,131]. In fact, loss of CALM disturbs the endosomal/autophagosomal system [111,123,131] which is in line with its ability to sort VAMP proteins that are critical for several intracellular trafficking events. In contrast to other endocytic adaptors CALM appears also to remain associated with its cargos after endocytosis and to guide their trafficking through the endosomal system. This was for instance reported for LRP1 [128].

### 2.5. HIP1 and HIP1R

The name of HIP1 originates from its initial identification as “Huntingtin-interacting protein” [134] since it binds the polyglutamine-containing protein Huntingtin that causes Huntington’s disease. HIP1R (HIP1 related; also known of HIP12) was later identified by structural homology [135,136]. While not binding to Huntingtin [135], HIP1R shares with HIP1 its association with the endocytic machinery [137]. HIP1 is enriched in the brain, but also found to some extent in peripheral tissues, especially also in reproductive organs [138]. HIP1R appears to be more widely expressed [139].

Like the other endocytic adaptors, HIP1 and HIP1R were both shown to localize to Clathrin-coated pits [140,141,142]. They share with AP180/CALM their N-terminal ANTH domain [29] which serves in AP180/CALM not only as a lipid interaction module, but also as a cargo binding domain (Figure 2). However, so far the HIP1/HIP1R ANTH domain has not been shown to bind to cargo even though HIP1 was reported to specifically facilitate the internalization of glutamate receptors [138,143]. In addition to the ANTH domain, HIP1 like most other endocytic adaptors contains motifs for binding to the Clathrin heavy chain and to AP-2 [141,142,144], but these motifs are poorly conserved in HIP1R [137]. However, it was discovered that the central coiled-coil domain does not only allow dimerization, but also mediates the interaction with Clathrin light chains [145] and thus stimulates Clathrin assembly [145].

Finally, both proteins contain a C-terminal Talin homology domain for interacting and potentially stabilizing F-Actin [146]. HIP1R in addition has a proline-rich domain that binds to the SH3 domain of the Actin regulator Cortactin [147]. Therefore HIP1/HIP1R are often not primarily viewed as cargo-specific endocytic adaptors, but might also promote endocytosis by connecting the Clathrin coat to the Actin organization in absence of HIP1R [148], while no endocytic or Actin alterations were detected in HIP1/HIP1R DKO mouse embryonic fibroblasts [149] which showed normal TfR uptake arguing again for a great resilience of the endocytic process based on the redundant assembly of endocytic factors.

#### 2.5.1. Lessons from HIP1- and HIP1R-Deficient Animal Models

Interestingly, in C. elegans loss of the homologous protein Hipr-1 led to a synaptic accumulation of Synaptobrevin, however it was not addressed whether this alteration is due to Hipr-1 acting like AP180/CALM as a specific sorter for Synaptobrevin or whether it is due to a general defect in the synaptic vesicle cycle [150].

In line with the normal TfR uptake observed in cells depleted of HIP1/HIP1R, the single mouse KO mutants of HIP1 and HIP1R did not show early lethality. In other respects the phenotypes of the several different KO mouse strains generated for HIP1 were quite divergent which was speculated to be due to the expression of undetected polymorphic alleles or due to differences in strain background. The least affected HIP1 KO strain suffered only from testicular degeneration [151], a phenotype also present in other HIP1 KO mouse lines which was later attributed to defects in spermatid maturation and decreased sperm motility [152]. A more affected HIP1 KO strain did not show alterations during the first weeks of life, but manifested progressive phenotypes from 3 months onwards including tremors and pronounced kyphosis culminating in the premature death of the animals [138]. A detailed electrophysiological characterization of this mouse line revealed a small decrease in long-term depression [143] in line with the decreased AMPA- or glutamate-triggered glutamate receptor uptake the authors reported [138] which appears to be the clearest case for a specific cargo sorting function of HIP1. In a later study glutamate receptor endocytosis was also found decreased upon NMDA (N-methyl-D-aspartate) stimulation [143]. Interestingly, HIP1 appears to interact directly with the NMDA receptor, and HIP1 loss protects neurons against NMDA-trigggered excitotoxicity [143]. However, it is not clear which effect HIP1 binding has on the NMDA receptor which has also binding sites for AP-2-dependent sorting. Further electrophysiological studies detected a slight increase in paired pulse facilitation, as observed also in AP180 KO mice [105], and a reduced recovery from synaptic depression in the more affected HIP1 KO mice [150] pointing to alterations in synaptic vesicle recycling and/or release. Finally, another HIP1 KO strain displayed in addition to the spinal defects and infertility hematopoietic alterations, micro-ophthalmia and cataracts [153]. The molecular basis for most of these observed phenotypes is still enigmatic.

The loss of HIP1R was first reported to have no effects [139], later HIP1R KO mice were shown to lose gastric parietal cells by apoptotic cell death leading to epithelial abnormalities [154]. Mice deficient for HIP1 and HIP1R showed profoundly aggravated phenotypes. They have for instance much earlier-onset kyphosis underlining the functional overlap between the two proteins [139]. The phenotypic alterations were rescued by expression of human HIP1 [149] thus proving the specificity of the phenotype. More recently, an interesting HIP1 KO mouse model with the option of conditional re-expression of a single copy of human HIP1 in specific tissues was generated, in order to unravel the tissue-specific function of HIP1 [155]. In contrast to the proposed role of HIP1 as sorter for glutamate receptors in the brain, this mouse model revealed that the selective expression of HIP1 under the brain-specific hGFAP promoter was not sufficient to rescue the presumably neurodegenerative phenotypes observed in HIP1 KO mice, while its expression in spleen, liver and kidney did rescue, thus arguing for an important role of HIP1 in these organs. Interestingly, the DKO mice were also shown to have low phosphocholine levels, however, it is presently enigmatic how this is linked to the phenotypic alterations [155]. Studies involving HIP1 KO mouse models also indicated that loss of HIP1 protects against arthritis, likely due to reduced invasiveness of synovial fibroblasts [156] and that HIP1 deficiency inhibits prostate tumorigenesis in vivo [157].

#### 2.5.2. Links of HIP1 to Human Disease

It is well established that HIP1 is overexpressed in a variety of human cancers including brain, colon and breast cancer as well as lymphoma [157,158,159,160]. HIP1’s association with EGFR was suggested to partially contribute to its oncogenic effect [161]. But also other non-endocytic functions of HIP1 such as its connection to apoptosis [162] or transcriptional regulation [163] might contribute. HIP1 is also often part of oncogenic fusion proteins [164,165,166]. Furthermore, a chromosomal microdeletion of HIP1 was linked to neurological deficits in human patients [167]. Finally, HIP1 is an interactor of the Huntington’s disease-causing Huntingtin protein, and this interaction is weakened in Huntingtin mutants suggesting a possible involvment of HIP1 in disease progression [134,168].

### 2.6. Ubiquitin Interacting Motif (UIM)-Containing Adaptors: Eps15 and Eps15R

Eps15 (EGFR pathway kinase substrate clone 15) was originally discovered due to its phosphorylation by the epidermal growth factor receptor (EGFR) [169]. Invertebrates contain only one gene for Eps15, while mammals acquired two functional paralogs during evolution, named Eps15 and Eps15R (Eps15-Related), also known as EPS15L1 (Eps15 like-1), sharing 41% identity and 61% similarity at the amino acid level [170]. From a structural point of view, Eps15/Eps15R display typical features of multidomain scaffolding proteins: Their N-terminal region contains Ubiquitin interacting motifs (UIMs), responsible for Ubiquitin recognition and monoubiquitination [171], and three EH (Eps15 homology) modules [172,173], evolutionary conserved structural domains which bind the amino acid motif NPF (asparagine-proline-phenylalanine) of target proteins such as Numb, Stonin2 and Epsins [174,175,176]. Their middle part harbors heptad repeats forming a coiled-coil structure important for Eps15 and Eps15R homo- and heterodimerization [177] and for binding to the endocytic scaffold Intersectin1/2 [178]. The C-terminal region comprises a proline-rich domain characterized by the presence of multiple DPF (aspartate-proline-phenylalanine) repeats, essential for the interaction with AP-2 [179] (Figure 2). In addition, Eps15R was shown to directly bind the Clathrin terminal domain [180]. In vitro studies, supported by structural data, first suggested that Eps15/Eps15R can form complexes with multiple endocytic proteins including AP-2, Dynamin, Intersectin1/2 and Stonin2, underlining their involvement in endocytosis [181,182].

In order to define the cellular functions of Eps15/Eps15R, many laboratories have used RNA interference approaches in diverse cell lines showing that these proteins act mostly in a redundant manner with other endocytic adaptors during constitutive Clathrin-mediated endocytosis [183]. Only when Eps15/Eps15R were depleted in conjunction with Intersectin1/2 there were effects on the recruitment of additional factors of the early endocytic module [85]. However, Eps15/Eps15R act in addition as specific adaptors for select cargo proteins. Cargo recognition occurs mostly via the binding of their UIM domain to Ubiquitin. Indeed, not only the ubiquitinated EGFR [184] is recognized in this manner by Eps15, but also the connexin 43 (Cx43) [185] and the glutamate receptor subunit GluA1 [186]. Instead, the hepatocyte growth factor receptor Met was shown to require the Eps15 coiled-coil domain for its sorting [187]. However, it is not clear yet whether these sorting events are physiologically relevant and whether Eps15R plays a role there as well. On the other hand, Eps15R was reported to act in the trans-endocytosis of EphB/ephrinB complexes [180]. The localisation of Eps15 at endosomal/TGN compartments [188,189] and the partial residence of Eps15R in the nucleus [190] suggest additional non-canonical functions for which the physiological importance is likewise unclear.

#### 2.6.1. Studies of Eps15 in Non-Mammalian Organisms

At the organism level, studies in C. elegans and D. melanogaster, where only one gene of the Eps15 family exists, indicate that Eps15 plays an essential role in the nervous system by regulating synaptic vesicle recycling. Indeed EHS-1 (Eps15 homologue sequence 1), the orthologue of Eps15 in nematodes, is enriched at synapses, and null mutants exhibit a depletion of synaptic vesicles resulting in impaired neurotransmission and locomotion [191]. In D. melanogaster, Eps15 is also broadly expressed in the nervous system and enriched at presynaptic sites. Under high-frequency stimulation, Eps15 mutant flies display an increase of abnormally large vesicles, a reduction in synaptic vesicle density and an inability to sustain neurotransmission [192,193]. In zebrafish, Eps15R was identified as a determinant of T cell development [194].

#### 2.6.2. Lessons from Eps15- and Eps15R-Deficient Mouse Models

In mammals, genetic duplication events increase the complexity and robustness of the endocytic network so that deletion of a single member often has no or a very mild phenotype. This is also the case for Eps15 in mice. Even though Eps15 is quite ubiquitously expressed, Eps15 KO mice are viable and fertile [195]. Moreover, primary mouse embryonic fibroblasts derived from Eps15 KO mice did not show impairments in TfR and EGFR endocytosis, likely due to functional redundancy with Eps15R or compensation by other endocytic proteins [195]. The only significant change in Eps15 KO mice identified so far was a subtle alteration in B cell lymphopoiesis leading to increased marginal zone B cell numbers, however the underlying mechanism is not understood [195]. Recently, Eps15 and Eps15R redundant and non-redundant functions were revealed via the generation of Eps15R KO and Eps15/Eps15R DKO mice [170]. In contrast to the lack of obvious defects in Eps15 KO mice, the deletion of the brain-enriched Eps15R leads to perinatal lethality with >50% of animals dying within the first two days, likely due to problems with respiration and feeding. The surviving animals show a reduced growth rate, alterations in behavioral tests and finally die at about 2 months old. Partially in agreement with the C. elegans and D. melanogaster null mutants, Eps15R KO hippocampal neurons display a reduction in synaptic vesicle density and, upon strong chemical stimulation, an increase in endosomal-like vacuoles. Intersectin1 levels were reduced in brain lysates, while all other tested endocytic and synaptic proteins were normal. In summary, these data indicate a non redundant role of Eps15R in neurons [170]. However, electrophysiological recordings, additional functional assays for perturbations in potential cargo proteins and an in depth electron microscopic analysis of both pre- and postsynaptic compartments are still needed to decipher the exact role of Eps15R in neuronal physiology.

Eps15/Eps15R DKO mice die around embryonic day 9.5 and display severe morphological and vascular defects at this stage demonstrating an important and redundant function of Eps15/Eps15R in embryonal development [170]. Conditional DKO mice with Eps15/Eps15R deletion in the hematopoietic system suffer from impaired red blood cell maturation and thus anemia [170], reminiscent of constitutive CALM KO mice [124]. As in the CALM KO mice, the anemia is a direct consequence of the defective endocytosis of TfR and the ensuing disruption of iron metabolism and haematopoiesis. Thus Eps15/Eps15R also have an important and redundant function in TfR endocytosis in the hematopoietic system [170].

#### 2.6.3. Involvement of Eps15 and Eps15R in Human Disease

Regarding human diseases Eps15 was originally identified as an oncogene which induces the transformation of NIH3T3 cells upon overexpression [169]. However, there is also a recent report stating that high Eps15 expression levels correlate with a favorable clinical outcome of breast cancer [196]. Deletions in the Eps15R gene have been linked to split-hand/split-foot malformation (SHFM) [197,198], showing once more an important role for Eps15R during development.

### 2.7. Epsin N-Terminal Homology (ENTH)- and UIM-Containing Adaptors: Epsins

The Epsins comprise three family members, Epsin1–3, with multiple roles in endocytosis, and a more distant relative, Epsin4 (also called EpsinR, Enthoprotin or Clint) with a function in endosomal transport which will not be described further here. Epsin1 and Epsin2 are rather ubiquitously expressed including high expression levels in the brain, while Epsin3 displays a more restricted expression pattern being present in keratinocytes upon wounding [199] and parietal cells in the stomach [200]. Epsin proteins share structural similarities with the AP180 and HIP1 protein family in starting off with an N-terminal membrane binding domain, the ~150 amino acid long Epsin N-terminal homology (ENTH) domain which mediates PI(4,5)P2 binding, followed by an unstructured C-terminus. As endocytic adaptors they harbor multiple NPF motifs for binding EH domain containing proteins like Eps15 [201], DPW motifs for binding to the AP-2α ear [201] and two interaction sites for Clathrin [202]. For cargo recognition they contain like Eps15/Eps15R Ubiquitin-interacting motifs (UIMs) [203] turning them into specific sorting proteins for ubiquitinated proteins (see below) (Figure 2).

Like AP180 family proteins Epsins were assumed to play a general role in Clathrin-mediated endocytosis because they do not only bind cargo, but also promote the assembly of Clathrin cages in vitro [204] and induce membrane curvature via the insertion of an amphipathic α-helix [205]. However, due to their redundancy, the importance of Epsins for Clathrin-mediated endocytosis as such turned out to be hard to prove. Single and even double loss of Epsins did affect certain cargos, but did not impair endocytosis in general [206,207]. Only the analysis of triple knockdown respectively KO cells finally uncovered a general impairment of endocytosis in the form of stalled Clathrin-coated pits and decreased Transferrin uptake [208,209]. The analysis of Epsin triple knockdown cells suggested their involvement in a late stage of Clathrin-mediated endocytosis by promoting membrane fission via membrane remodelling. Interestingly, the later studies in triple KO mouse embryonic fibroblasts uncovered a defective coupling between Clathrin coat generation and Actin polymerization, which prevented the proper invagination of Clathrin-coated pits. This might be caused by the decreased recruitment of Hip1R, which is known to link Actin to the endocytic machinery [209], or potentially also by the misregulation of the Actin regulator Cdc42, in line with earlier reports showing that yeast Epsins can interact with Cdc42 GTPase activating proteins (GAPs) [210]. Both possibilities should be investigated in future studies.

#### 2.7.1. Lessons from Epsin-Deficient Animal Models

The fact that Epsins are not only acting as cargo adaptors is underlined by the observation that the single D. discoideum Epsin lacks UIM motifs. However, mammalian Epsins which all harbor this Ubiquitin recognition module have also a physiologically important function as sorters for ubiquitinated cargos. Epsins have been suggested to sort ubiquitinated EGFR [207], ErbB3 [211], the GPCR protease-activated receptor 1 (PAR1) [206], and most importantly Notch ligands [212,213,214,215,216], VEGFR2/3 (vascular endothelial growth factor receptor) [217,218] and LRP1 [219] as animal models underlined.

In line with the redundancy between Epsin1-3 observed in cell cultures, mice deficient in only one of the Epsin genes do not display overt defects. However, the double KO of Epsin1/2 results in embryonic lethality at E9.5-10 [213] due to organogenesis defects resembling those observed upon Notch signaling impairments. Notch target genes were in fact downregulated in the DKO consistent with the prior association of Epsins with Notch signaling in D. melanogaster and C. elegans [215]. Studies in Drosophila revealed that the transmembrane Notch ligands of the DSL (Delta/Serrate/Lag-2) family have to be endocytosed in an Epsin-dependent manner [213,216,220,221] to be able to pull on the bound Notch protein, thereby exposing a cleavage site within Notch and enabling subsequent Notch signaling. Recently, impaired Notch signaling was also described for Epsin-depleted murine embryonic stem cells leading to neural differentiation defects reminiscent of Notch loss of function [212].

Tissue-specific Epsin1/2 DKO mice in vascular epithelium did not reveal defects under normal conditions, but displayed disorganized vasculature in tumors thereby restricting tumor growth [217]. This phenotype could be traced back to defective internalization of ubiquitinated VEGFR2 resulting in excessive VEGFR2 signaling and less productive angiogenesis. In fact, a reduction in VEGFR2 was sufficient to restore productive angiogenesis [218], and a peptide blocking the Epsin–VEGFR2 interaction was able to impair angiogenesis within tumors, thereby inhibiting their growth akin to the loss of Epsin1/2 [222]. Similarly, Epsins were shown to mediate the internalization of ubiquitinated VEGFR3. Aberrant VEGFR3 signaling in lymphatic endothelial-specific Epsin1/2 DKO mice was associated with defects in the lymphatic system [223]. Finally, Epsins in myeloid cells such as macrophages were shown to promote atherogenesis among others by downregulating ubiquitinated LRP1 [219]. Also the brain-specific loss of Epsins in a Nestin-cre based triple KO mouse line has severe effects leading to a decreased number of born animals, progressive motor dysfunction and premature death before one month of age. However, the critical mechanisms underlying these defects have not yet been addressed [209].

#### 2.7.2. Involvement of Epsins in Human Disease

The role of Epsins in human disease pertains mostly to tumorigenesis. Epsins were found upregulated in cancers of skin, lung and prostate [2]. In a xenograft model of prostate tumor growth, the deletion of Epsins decreased tumor growth and improved survival [2]. The described oncogenic mechanisms of Epsins appear to be mostly related to non endocytic functions. For example, Epsins were shown to bind the Wnt signal pathway effector Dvl2 thereby prohibiting its degradation. The decreased stability of Dvl2 in absence of intestinal Epsins antagonized colon cancer growth by reducing oncogenic Wnt signaling [224]. Another non canonical role of Epsins is their impact on mitotic [225] and meiotic spindle morphology [226] which was attributed in part to altered Cdc42 activity [226]. An important role of Epsins in cell division was confirmed later by the studies on triple KO mouse embryonic fibroblasts which display impaired cytokinesis [209].

### 2.8. PTB-Domain Containing Adaptors: ARH, Dab2, Numb and Numbl

ARH (autosomal recessive hypercholesterolemia), Dab2 (Disabled2) and Numb/Numbl (Numblike) are monomeric adaptor proteins that share an N-terminal phosphotyrosine-binding domain (PTB; also called phosphotyrosine interacting domain, PID) for cargo recognition. This PTB domain recognizes [FY]XNPX[YF] signals which were first identified in the LDLR [8], and have since been found in a number of transmembrane proteins. At the same time the PTB domain can interact with phosphoinositides [227]. In their C-terminal tails the adaptors harbor the usual motifs for binding to Clathrin and AP-2 [227,228,229] (Figure 2).

#### 2.8.1. The Role of ARH in Human Disease

ARH is the endocytic adaptor protein that is most closely linked to a human disease. Its name reflects the fact that mutations in ARH cause the metabolic syndrome “autosomal recessive hypercholesterolemia” [1]. Hypercholesterolemia is a risk factor for cardiovascular disease leading to early-onset coronary atherosclerosis in affected patients. The hypercholesterolemia arises from a defective clearance of cholesterol-carrying LDLs. These LDLs are mostly endocytosed in the liver after binding to the LDLR. Consistently, mutations in the LDLR also cause hypercholesterolemia. ARH binds to a NPXY motif in the LDLR [228] and facilitates its internalization.

#### 2.8.2. Phenotypes of ARH-Deficient and ARH/Dab2 DKO Mice

In ARH KO mice, the LDLR accumulates at the cell surface of hepatocytes, and the mice display like ARH patients increased cholesterol levels [230,231]. However, the cholesterol elevation is less pronounced than upon loss of the LDLR itself [230]. This finding together with the observation that some cell types such as fibroblasts do not need ARH for efficient LDLR endocytosis [1] argues for the existence of tissue-specific compensatory factors. In line with this, Dab2 was also found to bind to the NPXY motif of the LDLR. While the isolated loss of Dab2 only mildly affects serum cholesterol levels [232], the combined deletion of Dab2 and ARH increased the hypercholesterolemia to the level found upon LDLR loss of function [231] suggesting that Dab2 and ARH act in parallel in LDLR endocytosis. In fact, Dab2 and ARH have also overlapping functions in respect to the sorting of the LDLR family protein LRP2 [233] and AMN [234] as described in the section on Dab2.

In addition, ARH binds to a variant of the canonical NPXY motif within ROMK (renal outer medullary potassium channel), which is involved in the maintenance of the potassium balance [235]. The relevance of ARH for ROMK function was underscored by the fact that ARH KO mice display an altered ROMK response upon K^+^ intake [235]. In addition to its canonical role as endocytic adaptor ARH is implicated in centrosome assembly, mitosis and cytokinesis via its binding to centrosomal proteins [236] and spindle components [237]. In line with this, ARH KO mouse embryonic fibroblasts exhibit altered centrosomes, defective mitotic spindles, prolonged cytokinesis and slower growth [236,237].

#### 2.8.3. Functions of Dab2

Dab2 like ARH harbors motifs for binding to Clathrin and AP-2, but in addition it contains NPF motifs recognized by the EH domains of Eps15 and Intersectins [238]. In addition, Dab2 binds via its C-terminal serine- and proline-rich regions to the Actin-associated motor protein Myosin VI [239] thereby likely facilitating the generation or transport of Clathrin-coated vesicles (Figure 2). Of note, there are two splice variants of Dab2, and the shorter variant (p67) lacks a number of motifs for interacting with endocytic proteins [240,241]. Thus, it can only partly rescue endocytosis in isoform-specific knock-in mice [32].

Dab2 is related to the D. melanogaster protein Disabled [242] which has with Dab1 a second ortholog in mammals. While Dab2 is ubiquitously expressed [243,244], Dab1 is a brain-specific protein [245,246] mainly acting in neuronal positioning downstream of Reelin signaling. Consequently, there is no functional redundancy between Dab2 and Dab1, while a partial compensation by Numb and ARH was detected in Dab2 KO mice [231,232]. As mentioned, Dab2 shares functions with ARH in the endocytosis of the LDLR and also in the uptake of other family members which contain NPXY motifs such as LRP2. LRP2, also named Megalin, is a kidney-specific scavenger receptor mediating protein reuptake in the proximal tubule. Missorting of Megalin in absence of Dab2 leads to mildly increased protein levels in the urine (proteinuria) of conditional Dab2 KO mice [247] and likely plays a role in the embryonal lethality of constitutive Dab2 KO mice as detailed below.

LRP6, a co-receptor of LRP5 and Frizzled proteins which acts in the transduction of signals by Wnt proteins, is likewise bound by Dab2. In this case the interaction with Dab2 serves to reroute LRP6 from Caveolin-mediated endocytosis into the Clathrin-mediated endocytic pathway thereby inhibiting Wnt signalling [248]. ApoER2, another member of the LDLR family which plays as receptor for Reelin an important role in neuronal positioning, also harbors an NPXY motif which is recognized by Dab2 [249]. The same is true for the protein AMN which forms a complex with the multi-ligand receptor Cubilin which is for instance crucial for the dietary uptake of intrinsic factor-vitamin B12 in the intestine. AMN contains two NPXY motifs which bind in a redundant manner to Dab2 and ARH thereby facilitating its endocytosis [234]. Also the VEGFR is supposed to at least partially rely on Dab2 for its endocytosis [250].

Unbiased surface biotinylation studies in Dab2 depleted HeLa cells revealed the cell adhesion receptor Integrin β1 as another cargo for Dab2. Also here binding is mediated via an NPXY motif in the cytosolic tail of the cargo [238]. Localization studies suggested that Dab2 mediates the uptake of dispersed inactive Integrins rather than those localized within focal adhesions. Depletion of Dab2 led to decreased cell migration. For the efficient uptake of β1 Integrins Dab2 needs to be able to recruit the EH domain proteins Eps15 and Intersectin via its NPF motifs [238]. Dab2 is also involved in the uptake of Fibrinogen which is bound by the Integrin heterodimer αIIβ3 [251]. More recently, Dab2 as well as Numb were also shown to interact with Integrin β5 tails [252], however, independently of endocytosis (see Section 4).

CFTR (cystic fibrosis transmembrane conductance regulator) is an epithelial chloride channel which is important for the regulation of epithelial ion and water transport. Mutations in CFTR cause cystic fibrosis. Dab2 was revealed to play a role in CFTR endocytosis and also in its post-endocytic trafficking in human airway epithelial cells [253], but not in intestinal epithelial cells [254] highlighting the tissue-specificity of compensatory mechanisms.

Curiously, Dab2, in some situations, was demonstrated to facilitate endocytosis independently of AP-2. For example, in the absence of AP-2 it could still promote the uptake of CFTR in human airway epithelial cells [254], the internalization of Fibrinogen bound to αIIβ3 integrins [251] and the endocytosis of LDLR in HeLa cells [81]. Mulkearns et al. hypothesized that Dab2 might be able to internalize these cargos in absence of AP-2 by taking over the recruitment of additional endocytic factors otherwise recruited by AP-2. In fact, they revealed that the DPF motifs within Dab2 which normally interact with AP-2 can also bind the µHD of FCHO2. This interaction was crucial for allowing LDLR endocytosis in absence of AP-2 [81]. This suggests that Dab2 does not only serve as cargo-specific adaptor in conjunction with AP-2, but is able to take over AP-2’s role as major interaction hub within the endocytic network.

In addition to the interaction surfaces for cargo and endocytic factors, Dab2 comprises also a proline-rich domain (PRD) for interacting with SH3-containing signaling molecules such as Grb2 [244], Fyn, Src and Dvl [255] (Figure 2). Because of this, Dab2 has not only been classified as an endocytic adaptor, but also as a signaling adaptor [256] like its family member Dab1. In line with this, Dab2 was reported to influence a range of intracellular signaling cascades ranging from the Ras/MAPK pathway to TGFβ and Wnt signaling [255,257,258]. In addition, Dab2 was discovered to play roles in platelet physiology and immunology [259].

#### 2.8.4. Insights from Dab2-Deficient Mouse Models

The overall importance of Dab2 for mammalian life is obvious from the fact that homozygous Dab2 KO mice are embryonically lethal at a stage even prior to gastrulation [247]. This severe phenotype is attributed to a defect in the development of extra-embryonic endoderm likely caused by the missorting of adhesion molecules [260]. Both Megalin and E-cadherin were not restricted anymore to the apical surface of endodermal cells in Dab2 KO E5.5 embryos. These observations led to the proposition that Dab2 is required for their apical removal by endocytosis thereby promoting the establishment of apical-basal polarity, which appears to be a prerequisite for proper endoderm formation [261]. For Megalin this notion is well in line with the data on its Dab2-dependent internalization in proximal tubule cells [262]. For E-cadherin the assumption was not tested so far, and it is not clear, how Dab2 and E-cadherin might interact since E-cadherin does not have an obvious NPXY motif, however the two molecules share Myosin VI as binding partner which might bridge their interaction.

Conditional Dab2 KO mice that bypass the developmental defect show only mild phenotypes (proteinuria, increased serum cholesterol; for a comprehensive list please see [256]) and have a normal life span. The fact that the conditional KO mice that set in after the early developmental requirement of Dab2 do not show striking phenotypes reinforces the idea of functional redundancy with the related adaptors ARH and Numb which are often expressed in the same cell types.

#### 2.8.5. Links of Dab2 to Human Disease

The strongest link between Dab2 and human disease is its loss in many cancers such as ovarian, breast, colon, prostate, esophageal, nasopharyngeal and head and neck cancer [256] which is likely due to abnormal promoter hypermethylation [259]. In fact, a short mRNA fragment of Dab2 was initially identified due to its selective absence from ovarian cancer cell lines and was accordingly coined “differentially expressed in ovarian cancer” (DOC-2) [263]. In line with this, Dab2 heterozygous and homozygous mice also have a slightly reduced tumor incidence [264]. Loss of Dab2 seems to promote tumorigenesis while not being a sufficient insult on its own. The molecular basis for the tumor suppressive function of Dab2 are currently unclear since its suppression of Ras/MAPK signaling [256] or its impact on cell adhesion, cell migration and epithelial organization could all be imagined to influence tumor growth. Consequently, it is not known whether the loss of Dab2 in its capacity as endocytic adaptor or as signaling adaptor is most decisive for its role in tumorigenesis.

#### 2.8.6. Insights into Numb Function from D. Melanogaster

Numb was first studied in D. melanogaster sensory organs where it plays a crucial role in cell fate determination. During sensory organ development, cell fate is determined by the differential level of Notch signaling in each of the two daughter cells arising from asymmetric cell division. The difference in Notch signaling is triggered by the asymmetric distribution of Numb between the two daughter cells [265] which also translates into an asymmetric distribution of AP-2 [266]. In the Numb-positive daughter cell, the endocytosis of Notch is facilitated leading to an inhibition of Notch signaling [265]. This might be due to direct binding between Numb and Notch [267], or bridged via binding to Sanpodo, an interactor of Notch, which was shown to bind the PTB domain of Numb via a YTNPAF motif [268]. While this finding is well in line with a function of Numb as endocytic adaptor, its impact on Notch signaling appears to be more complex since Numb was also reported to influence the postendocytic trafficking [269] and degradation of Notch [33] as well as its ligand Dll4 [270]. In fact, Numb has been shown to interact not only with endocytic proteins and cargo, but also with components of the recycling machinery such as EFA6B-Arf6 [271] and EHD proteins [272] and with Ubiquitin ligases [33], and is implicated in the sorting of proteins such as N-cadherin [273] and APP [274].

#### 2.8.7. The Role of Numb im Mammals

The function of Numb as a determinant of neural cell fate is conserved in mammals [275]. However, mammals encode two related proteins: Numb and Numblike (Numbl) [33] which have overlapping functions. They do not only act in the brain, but also specify e.g., cardiac cell types [275]. In addition to Notch/Sanpodo there is a number of additional endocytic cargo proteins reported for Numb in mammals: Numb binds β1-Integrins via a classical PTB domain-NPXY interaction controlling their endocytosis specifically at the leading edge thereby modulating cell migration towards Integrin ligands [276]. Numb facilitates the endocytosis of E-cadherin by interacting with its binding partner p120 catenin [277] thereby playing a role in the maintenance of adherens junctions. It also recognizes a YVNHSF signal in the tail of the cholesterol binding protein NPC1L1 thereby triggering its endocytosis and the concomitant uptake of intestinal cholesterol [278]. Consequently, Numb deletion in murine intestine reduced dietary cholesterol uptake [278]. Moreover, Numb binds a YVNFFG motif in the excitatory amino acid transporter type 3 (EAAT3) thereby facilitating its endocytosis [279], and it controls the endocytosis of metabotropic glutamate receptor 1 (mGlu1) in Purkinje cells [280]. Additional reported cargos are the transmembrane receptor tyrosine kinase ALK (anaplastic lymphoma kinase) [281] and the receptor Boc (brother of CDON) which plays a role in axon guidance [282].

#### 2.8.8. Insights from Numb-Deficient Mouse Models

In line with its important role in cell fate specification constitutive Numb KO mice die at 11.5 suffering among others from severe defects in neural tube closure [283]. Numbl KO mice on the other hand do not have any severe defects [33]. However, the combined KO dies already at E9 confirming the partially overlapping function of the two proteins [284]. Neuron-specific DKO mice where the deletion sets in later have much milder effects ranging from anxiety-like behaviour (in case of deletions in the glutamatergic neurons in the dorsal forebrain [285]) to impaired motor coordination (in case of deletions in cerebellar Purkinje cells [280]).

#### 2.8.9. Involvement of Numb in Human Disease

Numb is closely linked to cancer. For example, breast cancers frequently display a loss of Numb expression. Not only does Numb’s regulation of cell fate decisions and proteins like Alk render Numb critical for tumorigenesis, but also its direct interaction with the important tumor suppressor p53 [286]. Numb binding to p53 prevents the degradation of the tumour suppressor. Consequently, breast cancer cells with a loss of Numb have at the same time decreased levels of the tumour suppressor p53 and increased activity of the oncogenic Notch signaling pathway [287]. Thus Numb’s endocytic function as well as its endocytosis-independent functions can contribute to cancer.

### 2.9. Arrestins—Adaptors for G Protein-Coupled Receptors

Arrestins are dedicated adaptors for the large family of G protein-coupled receptors (GPCRs) which comprises more than 800 members. Consequently, Arrestins have the largest known number of cargo proteins of any of the cargo-specific sorting adaptors. They were first discovered for their role in the phototransduction cascade where they are involved in the desensitization of the light-sensing GPCR Rhodopsin [288]. In fact, the desensitization of activated GPCRs via their phosphorylation by GPCR kinases (GRKs) and subsequent binding by Arrestins proved to be a general mechanism applying to virtually all GPCRs ranging from the β2-adrenergic receptor to the µ-opioid receptor.

There are two so-called visual Arrestins (Arrestin1 and 4) and two Arrestins operating outside the visual transduction cascade commonly known as β-Arrestin1 and β-Arrestin2 (alternative nomenclature: Arrestin2 and 3) which appear to bind to hundreds of different GPCRs [289]. One mechanism underlying GPCR desensitization is their Arrestin-triggered endocytosis [290]. For this β-Arrestins bind on the one hand to the Ser/Thr phosphorylated cytosolic GPCR tail via their N- and C-domain [291] and on the other hand to AP-2 via an RXR motif [292,293] and to Clathrin via an LIEF sequence [294] in their C-terminal tail (Figure 2). However, Arrestins do not only facilitate the endocytosis of GPCRs, but also prevent their interaction with G proteins thereby rapidly switching off G protein-dependent signaling, hence their name Arrestin for “arresting” signaling [288]. At the same time they can trigger G protein-independent signaling cascades and, like the related α-Arrestins [34], they can link GPCRs to Ubiquitin ligases. For a more detailed overview of their non endocytic functions that do not only relate to GPCRs, we refer the reader to a recent review [288].

#### 2.9.1. Insights from Arrestin-Deficient Mouse Models

Mouse models confirmed the importance of Arrestins for the regulation of GPCR signaling. Loss of Arrestin1 led to extended responses to light proving its physiological importance for Rhodopsin regulation [295]. A KO mouse model for β-Arrestin2 [296] also underlined its role in GPCR modulation, most prominently for the µ-opioid receptor whose desensitization was impaired leading to enhanced analgesia by its ligand morphine. Finally, mice deficient in β-Arrestin1 responded with increased cardiac contractility to agonists of the β2-adrenergic receptor [297]. However, in spite of the broad roles of GPCRs, the single β-Arrestin1 and β-Arrestin2 KO mice were viable and did not have severe phenotypes arguing for functional overlap in line with the fact that both proteins are more than 75% identical and widely co-expressed [298]. This notion was corroborated by experiments with double deficient cells which showed an aggravated impairment in β2 adrenergic receptor desensitzation [299] and by the analysis of β-Arrestin1/2 DKO mice which are embryonically lethal because of developmental defects [298].

#### 2.9.2. Links of Arrestins to Human Disease

Few disease-causing mutations in human Arrestins were identified so far. Most concern the visual Arrestin1 and lead to excessive Rhodopsin signaling and thus night blindness in Oguchi disease [300] and in some cases to retinal degeneration [301]. For the visual Arrestin4 no disease causing mutations are known. For the β-Arrestins there are likewise no disease-causing mutations identified yet, however some polymorphisms were reported which might be associated with neurological diseases [289]. Overall, it remains a challenge to understand how the small number of Arrestins can regulate the signaling of hundreds of GPCRs and even fulfill additional non-GPCR connected functions as signaling scaffolds.

### 2.10. Hrb and Hrbl

As the name Hrb (human immunodeficiency virus Rev binding protein; alternatively: hRIP, human Rev-interacting protein; RAB, REV/REC activation domain binding, AGFG1, Arf-GAP domain and FG repeats containing protein 2) implicates, this protein was initially not identified as endocytic adaptor but as binding partner for the protein Rev which is encoded by HIV [302,303,304] and promotes the nuclear export of viral RNAs. However, it was early on discovered that Hrb and the related 46% identical Hrb-like (Hrbl, encoded by the gene *AGFG2*) also interact with the endocytic protein Eps15 via NPF motifs [176,305] (Figure 2). Together they play a role in the Rev-dependent export pathway [305]. One of the endocytic roles assigned to Hrb and Hrbl also relates to HIV. This retrovirus downregulates the T helper cell protein CD4 via different mechanisms, one being recruitment of AP-2 by the viral protein Nef to promote the endocytosis of surface-localized CD4. Hrb/Hrbl, mostly via their interaction with Eps15, contribute to this HIV-induced CD4 internalization [306].

#### 2.10.1. Hrb as a Specific Sorter for VAMP7

However, more prominently, Hrb was identified as specific sorting adaptor for the SNARE protein VAMP7 (alternatively: TI-VAMP, tetanus neurotoxin-insensitive VAMP; SYBL1, synaptobrevin-like 1) [35] which facilitates e.g., lysosomal fusion events. In contrast to the shorter VAMPs such as VAMP2/3/8, VAMP7 contains a longer sequence stretch in front of its SNARE domain, the 120-140 amino acid comprising longin domain. This folded cargo domain becomes enwrapped by about 20 residues of the unstructured C-terminal region of Hrb. This is quite in contrast to most other cargo-adaptor interactions where a folded adaptor domain constitutes the recognition site for the cargo. Loss of Hrb causes a defect in the internalization of VAMP7 which leads to its ~2fold surface accumulation in normal rat kidney (NRK) cells, but not to a visible surface accumulation in HeLa cells suggesting cell-type specific redundant mechanisms [35] likely due to the expression of CALM (see Section 2.4). VAMP7’s longin domain is part of an autoinhibitory module and not accessible for Hrb binding while VAMP7 remains solitary. It only becomes exposed upon SNARE complex assembly assuring that only SNARE complexes, but not single VAMP7 molecules, are sorted for endocytosis by Hrb [35]. This is in contrast to the other two VAMP7 sorting adaptors CALM and AP180 which can only sort uncomplexed VAMP7 molecules since their binding to the VAMP7 SNARE domain is mutually exclusive with SNARE complex formation.

#### 2.10.2. Phenotypes of Hrb- and Hrbl-Deficient Mouse Models

Hrb and Hrbl are not yet associated with human diseases. In fact, also the mouse mutants have only mild respectively no defects. Male Hrb KO mice are infertile and display defective acrosome formation, probably due to a requirement for Hrb in the fusion of the proacrosomic vesicles during the process of acrosome biogenesis [307]. Hrbl KO mice are listed on the IMPC page as having no significant alterations (Hrbl KO phenotyping data. Available online: https://www.mousephenotype.org/data/genes/MGI:2443267). The generation of Hrb/Hrbl DKO mice might shed light on possible redundant functions in the future.

### 2.11. TTP/SH3BP4 and MACC1

Based on its endocytic function, the adaptor protein TTP was named “TfR trafficking protein” by the Di Fiore group [36]. However, its original name, which is still prevailing in the literature, is SH3BP4 for “SH3 domain binding protein 4” [308]. SH3BP4 is encoded in vertebrates, but not in lower organisms. In line with its Clathrin-binding site, AP-2 binding sequence and NPF motifs, TTP was shown to interact with Clathrin, AP-2 and Eps15 [36] (Figure 2). In addition, its SH3 domain is recognized by Dynamin. Pecularily, the same domain is also needed for the interaction of SH3BP4 with its cargo, the TfR. While this interaction was shown to be direct, the binding mode is still unclear since the TfR cytoplasmic tail does not harbor a canonical SH3 domain binding motif. Although Dynamin and TfR interact with the SH3 domain in a competitive manner, their binding was speculated to be non exclusive in vivo since SH3BP4 might be present as a dimer. SH3BP4 appears to be specifically enriched in TfR-positive Clathrin-coated pits and facilitates the endocytosis of the TfR, but not of other cargos like EGFR and LDLR. However, upon depletion of SH3BP4 not only TfR accumulated at the plasma membrane, but also Lamp1 [36]. While there are no further studies on SH3BP4’s role in endocytosis, it was later reported to be important for FGFR2b recycling [309]. It was also published to restrict the nuclear localization of β-catenin and thereby inhibit Wnt signaling thus promoting tumor development upon depletion [310]. SH3BP4 also acts as an inhibitor of the Rag GTPase complex which is important for mTORC1 activation downstream of amino acids [311]. Recently, it was also identified as novel pigmentation gene. The depletion of SH3BP4 decreased the melanin content of human melanocytes, however, the underlying molecular mechanism remains enigmatic [312]. The authors speculate that SH3BP4 could either modulate melanogenesis in its capacity as endocytic adaptor by influencing the trafficking of melanogenic enzymes or as a regulator of mTORC1 since mTORC1 signaling also impacts melanogenesis.

#### 2.11.1. Links of TTP/SH3BP4 to Human Disease

Apart from its promotion of tumorigenesis in mice, SH3BP4 has not been linked to disease. Even though a patient suffering from autism and intellectual disability was identified who has a genomic deletion encompassing SH3BP4 and AGAP1, it appears far more likely that loss of AGAP1 is the cause of the disease since this protein is known to play a role at synapses [313]. This would also be consistent with the fact that SH3BP4 KO mice display only an increased natural killer cell number according to the IMPC (SH3BP4 KO phenotyping data. Available online: https://www.mousephenotype.org/data/genes/MGI:2138297). Thus, SH3BP4 is not an essential gene, even though efficient TfR trafficking is crucial for life. However, this discrepancy is likely due to tissue-specific compensation by other factors. CALM is a prime candidate for an overlapping role within the haematopoietic system.

#### 2.11.2. MACC1, a Putative Endocytic Adaptor

Another candidate is MACC1 (metastasis-associated in colon cancer 1) which was identified in 2009 in a genome-wide search for genes that were differentially expressed between samples from healthy, colon cancer and metastatic tissue [314]. MACC1 has the closest similarity in domain structure and sequence to SH3BP4 and contains like SH3BP4 motifs for binding to endocytic proteins such as the clathrin box, NPF and DFP motifs. However, it is not clear presently whether MACC1 plays a role in endocytosis. It was mainly characterized as a driver of tumor metastasis via transcriptional regulation of genes that play a role in epithelial-mesenchymal transition and as facilitator of migration, invasion and proliferation [314].

## 3. One Cargo—Many Adaptors? Redundancy in Cargo Recognition

As evident from the preceding sections, there are many examples of cargo proteins that are sorted by more than one adaptor. E-cadherin is reported to be endocytosed with the help of AP-2, Dab2 and Numb [261,277,315]. VAMP2 can be sorted by AP180 and CALM [105], VAMP7 potentially by AP180, CALM [112] and Hrb [35]. The LDLR is sorted by Dab2 and ARH [231,316]. The most complicated cargo in terms of associated adaptors is certainly the TfR, likely also due to the fact that, being the prototype of a recycling receptor, many researchers have been studying its trafficking. Loss of CALM [113,124], Eps15/Eps15R [170], TTP [36], Dab2 [317] and Hrb [318] were all described to decrease its endocytic uptake, although the molecular determinants of the cargo/adaptor interaction are mostly unclear.

In principle, one cargo protein can interact with several adaptors via the same sorting motif like VAMP2. Alternatively, a cargo protein can also contain sorting motifs for different adaptors. Both possibilities can also be found combined adding an additional layer of complexity. An example for such increased complexity are the Integrins. On the one hand, they have more than one type of sorting motif. Indeed, Integrin heterodimers can have sorting motifs on their α- and β-subunits. β-subunits have NPXY-based sorting motifs, while a subset of Integrin α-chains contains a typical YXXΦ motif for interacting with AP-2 [319]. On the other hand their NPXY-based sorting motifs can interact with several PTB domain adaptors such as Numb, Dab2 and ARH [252].

Conversely, as apparent from the preceding paragraph, there are as many examples of adaptors sorting more than one cargo protein. AP-2 and the α-Arrestins are certainly the most prominent examples, but even the so-called cargo-specific adaptors are mostly pleiotropic having from two to several reported cargo proteins. In all these cases, it is largely unclear how the final decision is made which sorting motif and/or which adaptor prevails if there are multiple to choose from on each side. Some factors that will influence the outcome are the relative affinities of different cargo–adaptor pairs, their developmental and tissue-specific expression, their post-translational regulation and their subcellular localization which for example all differ between the closely related adaptors AP180 and CALM leading to divergent functions even though their cargo recognition module is highly similar. However, much is left to investigate in order to integrate the pleiotropic adaptor–cargo interactions into a coherent picture.

## 4. Endocytic Adaptors as Reticular Adhesion Components

A number of endocytic adaptors has been linked to focal adhesions, the Integrin-rich protein assemblies which connect the Actin cytoskeleton to the extracellular matrix and play a pivotal role in many important processes ranging from cell differentiation to migration. AP-2, Dab2, Numb and ARH all contribute to the sorting of Integrins [252,319] and were like Stonin1 [77] reported to facilitate the disassembly of focal adhesions. Recently, a structure long known as Clathrin plaque [320,321] or also flat Clathrin lattice [322] has gained new fame as reticular adhesion [321]. Reticular adhesions are mainly characterized by the presence of a specific type of Integrin heterodimer, αvβ5, and by the absence of most classical focal adhesion components such as Paxillin and Vinculin. Instead, reticular adhesions—being Clathrin plaques—are not only enriched in Clathrin but also in endocytic proteins, most prominently in endocytic adaptors. AP-2, Dab2, Numb, ARH, CALM, Eps15, Eps15R, HIP1 and HIP1R were all found within reticular adhesions [321].

The role of endocytic adaptor proteins within reticular adhesions is still rather unclear. A subset including ARH and Numb contributes to Integrin β5 recruitment [323]. The β5 Integrin cytosolic tail contains a typical NPXY motif, the recognition site for PTB-containing adaptors [252] like ARH, Numb and Dab2. In fact, ARH and Numb were both shown to bind the Integrin β5 tail via the NPXY motif. However, mutating this motif did not keep avβ5 from localizing in reticular adhesions, but the adhesions were fewer and smaller [323]. Likewise ARH knockdown led to a reduced number and size of Integrin β5 clusters [323]. In addition, Numb and Eps15/Eps15R contribute to β5 recruitment independent of the NPXY motif, possibly by interacting with ubiquitinated β5 tails. While ARH, Numb, Eps15/Eps15R (and possibly Dab2 which was not expressed in the tested cell type) all seem to act as linker between avβ5 and the Clathrin lattice, the importance of the remaining adaptors such as CALM is less clear. Are these proteins just there because of their binding to Clathrin and other endocytic factors? Or do they fulfill a specific function unrelated to their canonical role in endocytosis? For instance, do they help to cluster additional signaling factors at reticular adhesions?

The function of reticular adhesions itself is still only partially understood. They are believed to anchor cells during mitosis when focal adhesions are disassembled [324], were reported to act as signaling hubs [325,326] and appear to fulfill an important function in skeletal muscle by anchoring the sarcomere units to the plasma membrane [327,328]. The dissection of the physiological role of reticular adhesions is further complicated by the fact that β5 Integrin KO mice have a very limited phenotype [329] suggesting either redundancy with other Integrins or functions that are limited to very specific cell types and not essential for life.

## 5. Conclusions

**Figure 3:**
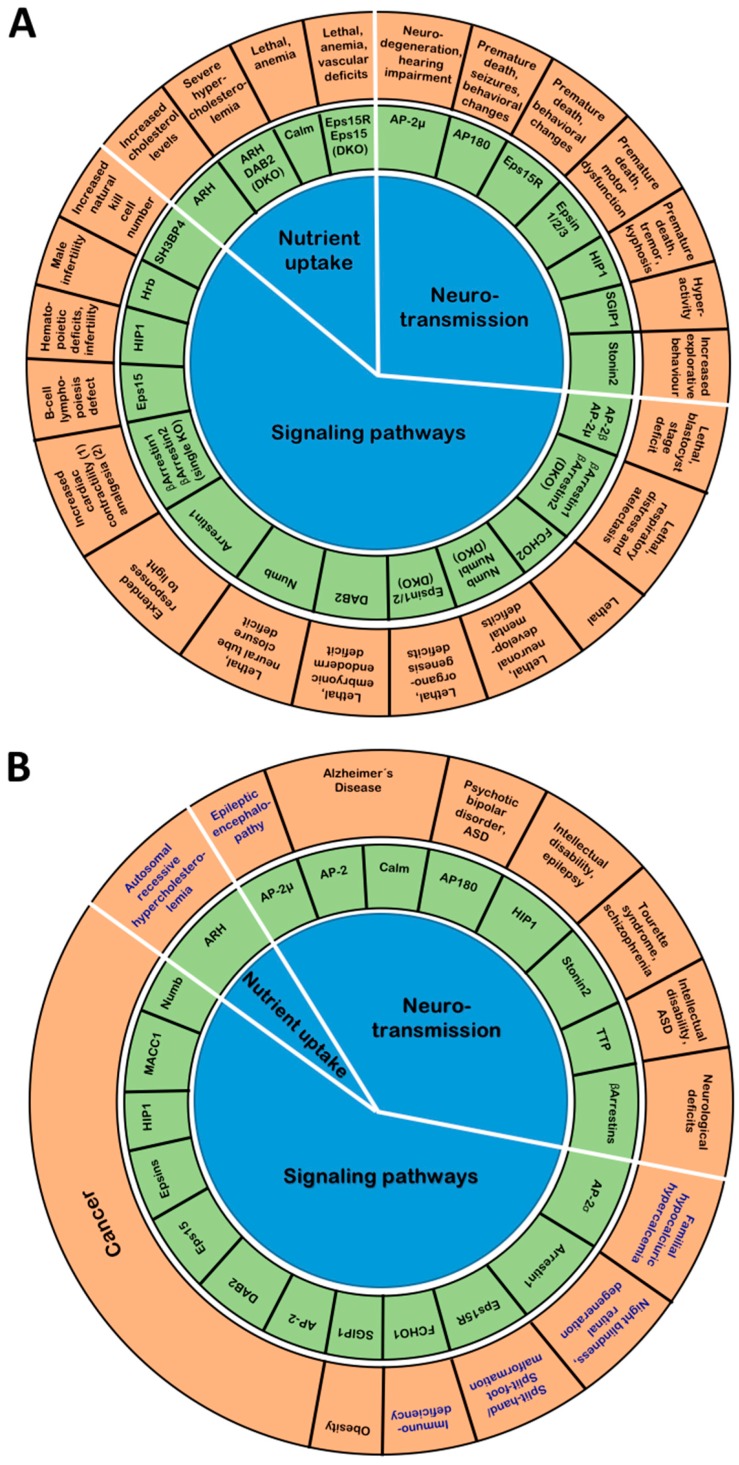
Endocytic adaptors, KO mouse phenotypes and human diseases. (**A**) Illustration of phenotypes due to deletion of endocytic adaptors in mice. (**B**) Illustration of human diseases associated with endocytic adaptors. Diseases caused by specific mutations in endocytic adaptors are highlighted in blue.

Endocytosis is crucial for life. Consequently, the loss of AP-2 which is not only a cargo adaptor but fulfills as central interaction hub a critical function in the organization of the endocytic machinery, is lethal at a very early embryonic stage. Also the other adaptor proteins do not exclusively act in the sorting of cargo, but contribute to endocytosis in multiple ways: They strengthen the cooperative low-affinity interaction network underlying coat assembly, they contribute to the activation of AP-2, they promote membrane remodeling, and they serve as connection to the Actin cytoskeleten. However, since the mammalian endocytic machinery has evolved into a highly redundant, largely fail-safe network of endocytic factors, the loss of a single endocytic adaptor is normally not critical. Therefore, mice deficient in a single adaptor protein are viable as long as the missorting of their specific cargo protein is compatible with life. In fact, the severity of the mouse phenotype or human disease resulting from the impaired function of a specific adaptor protein depends largely on the importance of the missorted cargo protein. Accordingly, the loss of AP180, which is needed for the sorting of the essential synaptic vesicle protein VAMP2, leads to premature death even though general endocytosis can still occur. Due to the large variety of cargo proteins, the observed diseases span from epilepsy to metabolical syndromes like hypercholesterolemia (Figure 3). In addition, since many endocytic cargos are signaling receptors which influence cell proliferation, migration and invasion, the deregulation of their trafficking itinerary contributes to the acquisition of proliferative and migratory capacities by cancer cells.

Endocytic adaptor proteins add to the growing list of proteins which are discovered to have more than just the originally identified “canonical” function. This is, for example, evident in neurons. Here, prior to endocytosis, endocytic adaptors help to confine newly exocytosed synaptic vesicle proteins [330]. In addition, the adaptors do not act exclusively at the plasma membrane. In fact, synaptic vesicles were discovered to be partially retrieved in a Clathrin-independent manner [331]. This Clathrin-independent endocytosis leads to the formation of large endosomal-like vacuoles from which synaptic vesicles are then regenerated with the help of Clathrin, AP-2 and AP180 as demonstrated by the accumulation of endosomal-like vacuoles upon depletion of either protein [12,105,331]. Finally, many endocytic adaptors have functions completely unrelated to endocytosis as exemplified by the role of AP-2 in the transport of autophagosomes. These additional non canonical functions complicate the dissection of the molecular mechanisms underlying adaptor-associated diseases. This is especially true for those adaptors that also act as signaling scaffolds and are associated with cancer since defects in the trafficking of signaling receptors and in the organization of signaling platforms are an equally likely cause of oncogenic alterations in signal transduction cascades.

Consistent with their important function endocytic adaptors are subject to complex regulation, often via multiple ubiquitination and phosphorylation sites, with the posttranslational modifications frequently being correlated with important cellular processes such as mitosis and synaptic vesicle cycling. Unfortunately, it was beyond the scope of this review to delineate these intricate regulatory layers which are an important field for future studies. Also the regulation of endocytic adaptors by epigenetics and alternative splicing remains largely unexplored so far, but is likely to have disease implications.

KO mouse models and the analysis of human mutations have already greatly advanced our understanding of the physiological role of endocytic adaptor proteins. With the advent of ever more powerful approaches to find rare disease mutations and disease-associated SNPs we will certainly witness an increased association of endocytic adaptors with human diseases in the future. To understand the molecular mechanisms underlying adaptor-associated diseases, further studies in model organisms will be instrumental. Especially in vivo studies of specific mutations targeting interaction surfaces or sites of posttranslational modifications will be essential to disentangle the contribution of endocytic and non canonical roles of endocytic adaptor proteins and to understand the relevance of their intricate posttranslational modification for their function. 

Detailed knowledge about adaptor-cargo interactions will be of high therapeutic value. To be able to selectively modify the trafficking of disease-relevant surface proteins will for example open up new avenues for targeted cancer therapies. A prominent example is the use of peptides blocking the Epsin-VEGFR2 interaction to impair tumor angiogenesis which inhibits tumor growth and metastasis in mouse models [332,333,334]. The selective inhibition of endocytosis could also prove useful in combination with cancer therapies, which exploit the antibody-based recognition of surface-localized tumor markers such as EGFR for the delivery of cytotoxic drugs. Increasing the surface level of the respective tumor marker would improve the efficiency of the therapeutic approach.

## Figures and Tables

**Table 1:** Overview of cargo recognition and physiological relevance of endocytic adaptors. Abbreviations are explained in the list of abbreviations.

Adaptor (Gene)	Signal–Adaptor Domain	Cargos	Endocytic Interactors	Mouse Phenotypes	Links to Human Disease
AP-2 (*AP2A1,* *AP2A2,* *AP2B1* *AP2M1,* *AP2S1*)	YXXΦ-µ2; [DE]XXXL[LI]-α/σ2; C2 domains –µ2	APP [335], ATP6V0A1 [336], ATP6V1H [337], BACE1 [338], CD4 [339], CD63 [340], CFTR [341], CL-P1 [342], CTLA-4 [343], CXCR2 [344], Cx43 [345], EAAC1 [346], EGFR [347], E-Syt2 [348], Frizzled4 [349], GABAAR [350], GLUT4 [351], GLUT8 [352], GluR2 [353], α2/α4 Integrins [319], KCC2 [354], Kir2.3 [355], L1 [356], Lamp1 [340], Lamp2 [340], LDLR [357], MHC-II [358], N-cadherin [359], NCKX2 [360], NR2B [361], Otoferlin [12], PAR4 [362], P2 × _4_ R [363], SorCS1a [364], SorCS1c [364], Synaptotag-min1 [60], TGF-β R [365], TGN38 [366], TfR [9], VGAT [367], VGLUT1 [368] etc.	Amphiphy-sin, Apache, AP180, ARH, β-Arrestins, CALM, Clathrin, DAB2, Epsins, Eps15/R, FCHO1/2, HIP1, Hrb, Intersectin, MACC1, NECAP1, Numb, Numbl, PI(4,5)P_2_, SGIP1, Stonin1/2, Synaptoja-nin, TTP etc.	Constitutive *AP2M1* KO: embryonic lethality [39]; constitutive *AP2B1* KO: perinatal lethality and cleft palate [40]; *AP2S1* del17 mouse: embryonic lethality; neuron-specific *AP2M1* KO: neurodegeneration and premature death [38]; IHC-specific *AP2M1* KO: hearing deficit [12]	*AP2M1* mutation: epileptic encephalopathy [3]; *AP2S1* mutation: familial hypocalciuric hypercalcemia type 3 [43]; AP-2α, AP-2β, AP-2σ downregulated in gliomas [48]; *AP2A1*, *AP2A2* association with AD [47]
AP180 (*SNAP91*)	SNARE motif-ANTH	VAMP2 [121,122]	AP-2, Clathrin, PI(4,5)P_2_	Constitutive KO: behavioral alterations, epileptic seizures, premature death [105]	Link to psychotic bipolar disorder [125] and to ASDs [126]; downregulated in gliomas [48]
ARH (*LDLRAP1*)	[FY]XNPX[YF]-PTB	Amnionless [234], β5 Integrin [323], LDLR [8], LRP2/Mega-lin [233], ROMK [235]	AP-2, Clathrin, PI(4,5)P_2_	Constitutive KO: increased cholesterol levels [230,231], altered ROMK response [235]; ARH/Dab2 DKO: pronounced hypercholesterolemia [256]	Mutated in Autosomal recessive hypercholestero-lemia (ARH) [1]
Arrestin1 (*SAG*)	pSer/pThr in GPCR-NT	Rhodopsin [295]		Constitutive KO: extended response to light [295]	Mutated in Oguchi syndrome: night blindness and sometimes retinal degeneration [300]
Arrestin4 (*ARR3*)	pSer/ pThr in GPCR-NT	Cone opsins [369]		Constitutive KO: diminished visual acuity and contrast sensitivity [369]	
β-Arres-tin1 = Arrestin2 (*ARRB1*)	pSer/ pThr in GPCR-NT	Hundreds of GPCRs, e.g. β2-adre-nergic receptor [290]	AP-2, Clathrin	Constitutive KO: altered µ-opioid receptor signaling causing enhanced morphine analgesia [297]; DKO with β-arrestin2: embryonic lethality due to developmental defects [298]	Polymorphisms of unclear significance linked to neurological diseases [289]; somatic mutations in breast cancer [370]
β-Arres-tin2 = Arrestin3 (*ARRB2*)	pSer/ pThr in GPCR-NT			Constitutive KO: altered cardiac β-adrenergic receptor signaling causing increased cardiac contractility [296]	
CALM (*PICALM*)	SNARE motif-ANTH	APP [129], Aβ-bound LRP1 [128], Nicastrin [131], TfR [113,124], VAMP2/3/4/7/8 [110,112,122]	AP-2, Clathrin, PI(4,5)P_2_	Constitutive KO: anemia, embryonal or perinatal lethality [113]; adult onset KO: viable [113,124]	SNPs in AD [127]; somatic mutation/gene fusion in ALL and AML [104]
Dab2 (*DAB2*)	[FY]XNPX[YF]-PTB	Amnionless [234], ApoER [249], CFTR [253], E-Cadherin [260], EGFR [256], FGFR [256], β1/β5 Integrin [276], LDLR [81], LRP1 [256], LRP2/Mega-lin [233], LRP6 [248], VEGFR [250]	AP-2, Clathrin, Eps15, FCHO2, Intersectin, MyosinVI, PI(4,5)P_2_	Constitutive KO: lethal prior to gastrulation [247]; conditional ubiquitous KO: mild proteinuria, mild increase in serum cholesterol, reduced tumor incidence [231,232,247,256,264] (see also [256] for complete list)	Downregulated in cancers (bladder, breast, colorectal, oesophageal, ovarian, prostate) [243,256,263,371,372,373,374]
Eps15 (*EPS15*)	Ubiqui-tin-UIM; CC-Met	Met [187], TfR [170], Ub-Cx43 [185], Ub-EGFR [184], Ub-GluA1 [186]	AP-2, CHC, Dynamin, Epsins, Intersectin, Numb, Stonin2, Synaptoja-nin	Constitutive KO: altered B cell lymphopoiesis [195]	Somatic mutations/gene fusion in AML and lung cancer [370,375]
Eps15R (*EPS15L1*)	Ubiqui-tin-UIM	EphB2/ ephrinB [180], TfR [170]	AP-2, CHC, Dynamin, Epsins, Intersectin, Numb, Stonin2, Synaptoja-nin	Constitutive KO: pre-/postnatal lethality, problems with respiration and feeding, growth deficits, behavioral alterations [170]; Eps15/Eps15R DKO: embryonic lethality, anemia, vascular defects [170]	Gene deletion causing SHFM
Epsin-1 (*EPN1*)	Ubiqui-tin-UIM	Ub-EGFR [207], ErbB3 [211], Notch ligands [212,213,214,215,216], LRP1 [219], PAR1 [206], VEGFR2/3 [217,218]	AP-2, Clathrin, Eps15, HIP1R, PI(4,5)P_2_	Single KOs: no effect; DKO: embryonic lethality [213]; Vascular endothelium- specific DKO: disorganized tumor vasculature; brain- specific DKO: less animals born, progressive motor dysfunction, premature death [217]	Upregulated in cancer [2,224,376]
Epsin-2 (*EPN2*)					
Epsin-3 (*EPN3*)	Ubiqui-tin-UIM		AP-2, Clathrin, Eps15, PI(4,5)P_2_	Constitutive KO: no effect	
FCHO1 (*FCHO1*)	Not known-µHD	Alk8 (zebrafish) [87] Mid2 (yeast) [26]	AP-2, Dab2, Eps15, PIs	No KO mouse reported	Mutated in combined immuno-deficiency [94]; downregulated in gliomas [48]
FCHO2 (*FCHO2*)	Not known-µHD			KO mouse at IMPC: preweaning lethality	
HIP1 (*HIP1*)		AMPAR [143]	AP-2, CHC, CLC, F-Actin	Constitutive KO (multiple lines): progressive tremor, ataxia, kyphosis culminating in premature death [138], decreased LTD [143], testicular degeneration [151], haematopoietic alterations, ophthalmic defects [153], partial protection against arthritis [156] and prostate tumorigenesis [157]; effects aggravated in HIP1/HIP1R DKO [139]	Chromosomal microdeletion causing neurological deficits [167]; overexpression/ somatic mutations/gene fusion in diverse cancers [157,158,159,160,166,370]; potential involvement in HD [134,168]
HIP1R (*HIP1R*)			CLC, Cortactin, F-Aktin	Constitutive KO: loss of gastric epithelial cells, epithelial abnormalities [154]	Overexpressed in colon cancer and CLL [377,378]
Hrb (*AGFG1*)	VAMP7 longin domain-CT un-struc-tured domain	VAMP7 [35]	Eps15	Constitutive KO: infertile due to defective acrosome formation [307]	
Hrbl (*AFGF2*)			Eps15	KO mouse at IMPC: no alterations	
MACC1 (*MACC1*)			AP-2, Clathrin		Upregulated in metastatic tissue, cancer-related SNPs [314]
Numb (*NUMB*)	[FY]XNPX[YF]-PTB	Alk [281], Boc [282], EAAT3 [279], E-Cadherin [277], β5/β1 Integrin [276], NPC1L1 [278], mGlu1 [280], Sanpodo/ Notch [267]	AP-2, Clathrin, PI(4,5)P_2_	Constitutive KO: embryonal lethality at E11.5 due to defect in neural tube closure [283]; different conditional KOs: reduced cholesterol upake [278], alterations in behaviour [285] and motor coordination [280]	Downregulated in breast cancer [379]
Numbl (*NUMBL*)	[FY]XNPX[YF] –PTB	?	AP-2, Clathrin, PI(4,5)P_2_	Constitutive KO: no severe defects [33]; Numb/Numbl DKO: embryonic lethality [284]	
SGIP1 (*SGIP1*)	C2A-µHD	Synaptotag-min1 [92]	AP-2, Dab2, Endophilin, Eps15, Intersectin, PIs, PS	KO mouse at IMPC: abnormal behaviour, cardiovascular phenotype etc.	Associations with obesity, EEG and ECG abnormalities to be confirmed [98,99,100,101]
Stonin1 (*Ston1*)	Not known-µHD	No directly interacting cargo known	AP-2	Constitutive KO: no obvious phenotype [77]	Upregulated in gliomas [48]
Stonin2 (*Ston2*)	C2A –µHD	Synaptotag-min1 [63,66]	AP-2, Eps15, Intersectin	Constitutive KO: behavioral and electrophysiological changes; no aggravation of defects in Stonin1/2 DKO [73]	Association with schizophrenia [79], lies within region mapped for Tourette disorder spectrum [78]
TTP (*SH3BP4*)	Not known-SH3 domain	Lamp1 [36], TfR [36]	AP-2, Clathrin, Dynamin, Eps15	KO mouse at IMPC: increased NK cell number; intestine-specific KO: increase in intestinal stem cells [310]	Deleted together with *AGAP1* in ASD/intellectual disability patient [313]

## Abbreviations

Aβ	Amyloid β
AD	Alzheimer’s disease
ALL	Acute lymphoblastic leukaemia
AML	Acute myeloid leukaemia
AMPA	α-amino-3-hydroxy-5-methyl-4-isoxazolepropionic acid
AMPAR	AMPA receptor
ANTH	AP180 N-terminal homology domain
APA	AP-2 activation domain
AP-2	Assembly protein 2
ArfGAP	Arf GTPase activating protein
ARH	Autosomal recessive hypercholesterolemia
ASD	Autism spectrum disorder
BAR	Curved domain named after Bin, Amphiphysin, Rvs
BDNF	Brain-derived neurotrophic factor
CC	Coiled coil domain
CHC	Clathrin heavy chain
CMML	Chronic myelomonocytic leukaemia
CLC	Clathrin light chain
CLL	Chronic lymphocytic leukaemia
CT	Carboxy-terminus
DD	Death domain
DKO	Double knock-out
ECG	Electrocardiagram
EEG	Electroencephalogram
EGF	Epidermal growth factor
EGFR	Epidermal growth factor receptor
EH	Eps15 homology domain
ENTH	Epsin N-terminal homology domain
F-BAR	FCH-BAR domain
FHH	Familial hypocalciuric hypercalcemia
GAP	GTPase activating protein
GPCR	G protein-coupled receptor
GTP	Guanosine triphosphate
HCV	Hepatitis C Virus
HD	Huntington’s disease
HIV	Human Immunodeficiency Virus
IDRs	Intrinsically disordered protein regions
IMPC	International mouse phenotyping consortium
iPSCs	Induced pluripotent stem cells
LDL	Low density lipoprotein
LDLR	Low density lipoprotein receptor
LTD	Long term depression
MP	Membrane lipid binding domain
µHD	µ-Homology domain
NK cells	Natural killer cells
NMDA	*N*-methyl-d-aspartate
NT	Amino-terminus
KO	Knock-out
LTD	Long term depression
SHD	Stonin homology domain
PI	Phosphoinositide
PI(4,5)P_2_	Phosphatidylinositol(4,5)bisphosphate
PRR	Prolin-rich region
PS	Phosphatidylserine
PTB	Phosphotyrosine binding domain
PTH	Parathyroid hormone
SHFM	Split-hand/split-foot malformation
SH3	Src homology 3 domain
SNARE	Soluble *N*-ethylmaleimide sensitive factor attachment protein receptor
SNP	Single nucleotide polymorphism
Talin-like	Actin binding domain
TfR	Transferrin receptor
TGFβ-R	Transforming growth factor b receptor
TrkB	Tropomyosin receptor kinase B
Ub	Ubiquitin
UIM	Ubiquitin interacting motifs
UPA	Domain named after Unc5, PIDD and Ankyrins
VAMP	Vesicle-associated membrane protein
VEGFR	Vascular endothelial growth factor receptor
ZU5	Domain named after tight junction protein ZO-1 and C. elegans Unc5
